# Statistical Inference for High-Dimensional Heteroscedastic Partially Single-Index Models

**DOI:** 10.3390/e27090964

**Published:** 2025-09-16

**Authors:** Jianglin Fang, Zhikun Tian

**Affiliations:** College of Science, Hunan Institute of Engineering, Fuxing Road, Xiangtan 411104, China; 70041@hnie.edu.com

**Keywords:** partially linear single-index models, high-dimensional data, oracle property, penalized empirical likelihood

## Abstract

In this study, we propose a novel penalized empirical likelihood approach that simultaneously performs parameter estimation and variable selection in heteroscedastic partially linear single-index models with a diverging number of parameters. It is rigorously proved that the proposed method possesses the oracle property: (i) with probability tending to 1, the zero components are consistently estimated as zero; (ii) the estimators for nonzero coefficients achieve asymptotic efficiency. Furthermore, the penalized empirical log-likelihood ratio statistic is shown to asymptotically follow a standard chi-squared distribution under the null hypothesis. This methodology can be naturally applied to pure partially linear models and single-index models in high-dimensional settings. Simulation studies and real-world data analysis are conducted to examine the properties of the presented approach.

## 1. Introduction

Consider the following partially linear single-index model (PLSIM)

(1) $$Y_{i}=\theta^{⊤}X_{i}+g\left(Z_{i}^{⊤}\gamma \right)+\varepsilon_{i},\;\;\;E\left(\varepsilon_{i}|X_{i},Z_{i}\right)=0\;\;\;i=1,\cdots ,n,$$

where $X_{i}\in R^{p}$ and $Z_{i}\in R^{r}$ are covariates, g(·) denotes an unknown function, $Y_{i}$ is a response variable, $\theta \in R^{p}$ and $\gamma \in R^{r}$ are parameter vectors, and $\varepsilon_{i}$ is an independent random error. Let $\mathrm{Var}\left(\varepsilon_{i}|X_{i},Z_{i}\right)=v\left(X_{i},Z_{i}\right)>0$, where the function v(X,Z) expresses potential heteroscedasticity. For model (1), the scale of the index parameter γ is generally not uniquely identifiable. That is, if the index parameter is multiplied by a nonzero constant while the nonparametric function is divided by the same constant, the model’s predictions remain unchanged. To ensure identifiability and avoid non-unique representations, it is standard practice to impose an identifiability constraint on the index parameter γ, for example, fixing one of its components to 1 or restricting it to have unit norm. We fix the first component of γ to 1 and denote the remaining components of *Z* as $Z_{-1}$. Model (1) is a combination of a partially linear model (PLM) and single-index model (SIM). As far as we know, Carroll et al. [1] introduced PLSIMs and developed a backfitting algorithm for estimation in their generalized form. Since their introduction, they have attracted much research attention, and extensive research has focused on estimating g(·) and the unknown parameter vectors θ and γ. For PLSIMs, Yu and Ruppert [2] showed a spline estimation approach via the penalty function; Zhu and Xue [3] proposed an empirical likelihood (EL) method; Xia and Härdle [4] introduced a semiparametric estimation procedure; Liang et al. [5] introduced an estimation method using profile least squares; etc.

In recent years, the analysis of high-dimensional data has evolved into a major frontier of statistical research, with applications spanning internet portals, hyperspectral imagery, financial applications, and high-throughput genomic data analysis within computational biology. See, e.g., Ma and Zhu [6], Fang et al. [7], Hao and Yin [8], Liu et al. [9], etc. Ma and Zhu [6] introduced efficient estimators for heteroscedastic PLSIMs allowing high-dimensional setting. While their methodology accommodated high-dimensional covariates, it restricted their dimensions to be fixed rather than allowing them to diverge as the sample size increases. Fang et al. [7] proposed EL estimators for high-dimensional PLSIMs, permitting the covariate dimension to diverge as the sample size increases.

In high-dimensional analyses, PLSIMs suffer from the inclusion of irrelevant covariates, leading to inefficient parameter estimators and reduced prediction accuracy. Pruning these non-informative variables from the sparse true model is thus a natural imperative. This has motivated the proliferation and rigorous study of various variable selection methods in contemporary statistics. Various variable selection methods have been investigated, including AIC and BIC (Breiman [10]), the LASSO penalty (Tibshirani [11]), the SCAD penalty (Fan and Li [12]), and so on. For PLSIMs, several variable selection methods were proposed. Xie and Huang [13] proposed a variable selection method for PLMs, which are special cases of PLSIMs, and proved that this method possesses the oracle property. Wang and Zhu [14] established nearly necessary and sufficient conditions for estimator consistency in SIMs under high-dimensional (“large *p* small *n*”) settings, which are special cases of PLSIMs. Zhang et al. [15] developed a method for variable selection and parameter estimation in high-dimensional PLSIMs. Lai et al. [16] studied variable selection for heteroscedastic PLSIMs by using the efficient score function. These methods, however, are largely constrained to scenarios where the covariate dimensions remain fixed.

Within nonparametric frameworks, Owen [17] developed the EL method for statistical inference. This approach retains likelihood methodology while eliminating parametric distributional assumptions. Since this method was proposed, it has been successfully extended to various circumstances, including linear models [18], generalized linear models [19], heteroscedastic PLMs [20], SIMs [21], and network data [22], among others. Chen et al. [23] demonstrated that EL remains valid when the data dimension diverges. In high-dimensional data settings, Tang and Leng [24] studied a variable selection method by penalized empirical likelihood (PEL) for linear regression models, and Leng and Tang [25] investigated a PEL method for general estimating equations. To our knowledge, applications to heteroscedastic PLSIMs have been scarcely explored, especially for variable selection in high-dimensional settings.

Empirical likelihood is a data-driven, nonparametric methodology that retains the merits of parametric likelihood while offering robustness and flexibility in incorporating auxiliary information to obtain estimates and construct confidence sets. Motivated by the PEL method for high-dimensional estimating equations in Tang and Leng [25], we aim to explore a variable selection approach using PEL for a PLSIM in a heteroscedastic high-dimensional situation, where dimensionality p→∞ and r→∞, as n→∞. For model (1), the PEL ratio is constructed using semiparametric efficient estimating equations, incorporating the semiparametric efficient score for the heteroscedastic PLSIM. We prove that PEL has the oracle property and excels at generating sparse models without requiring a prespecified parametric likelihood. Although existing variable selection techniques (e.g., Lai et al. [16]) also attain the oracle property, specifying a high-dimensional distribution remains theoretically challenging. Furthermore, the PEL ratio statistic satisfies Wilks’ theorem, converging to a chi-squared distribution under some regularity conditions, which facilitates hypothesis testing and produces range-respecting confidence regions. As a robust alternative to parametric likelihood ratios in high-dimensional settings, PEL combines the adaptive ability and statistical efficiency inherent in nonparametric likelihood methods, complementing the existing methods.

The rest of this article is organized as follows. Section 2 outlines methods of variable selection, parameter estimation, and asymptotic properties for high-dimensional heteroscedastic PLSIMs using PEL. Section 3 extends the method to PLMs and SIMs as special examples. In Section 4, we exhibit simulation results, and an application of the proposed method is stated in Section 5. Lemmas and technical proofs are shown in Appendix A.

## 2. Penalized Empirical Likelihood for PLSIM

Denote the weight function as $w\left(X_{i},Z_{i}\right)={\left[E\left(\varepsilon_{i}^{2}|X_{i},Z_{i}\right)\right]}^{-1}$, i=1,⋯,n. Using the kernel function $K_{h}\left(u\right)=h^{-1}K\left(u/h\right)$ with bandwidth h→0, the nonparametric estimators are defined as follows:

$$\begin{array}{ccc} \hat{E}\left\{\hat{w}\left(X,Z\right)|Z_{i}^{⊤}\gamma \right\} & = & \frac{\sum_{i\neq j}K_{h_{3}}\left(Z_{i}^{⊤}\gamma -Z_{j}^{⊤}\gamma \right)\hat{w}\left(X_{i},Z_{i}\right)}{\sum_{i\neq j}K_{h_{3}}\left(Z_{i}^{⊤}\gamma -Z_{j}^{⊤}\gamma \right)},\\ \hat{E}\left\{\hat{w}\left(X,Z\right)Z_{-1,i}|Z_{i}^{⊤}\gamma \right\} & = & \frac{\sum_{i\neq j}K_{h_{3}}\left(Z_{i}^{⊤}\gamma -Z_{j}^{⊤}\gamma \right)\hat{w}\left(X_{i},Z_{i}\right)Z_{-1,i}}{\sum_{i\neq j}K_{h_{3}}\left(Z_{i}^{⊤}\gamma -Z_{j}^{⊤}\gamma \right)},\\ \hat{E}\left\{\hat{w}\left(X,Z\right)X|Z_{i}^{⊤}\gamma \right\} & = & \frac{\sum_{i\neq j}K_{h_{3}}\left(Z_{i}^{⊤}\gamma -Z_{j}^{⊤}\gamma \right)\hat{w}\left(X_{i},Z_{i}\right)X_{i}}{\sum_{i\neq j}K_{h_{3}}\left(Z_{i}^{⊤}\gamma -Z_{j}^{⊤}\gamma \right)}, \end{array}$$

$$\hat{w}\left(X_{i},Z_{i}\right)=\sum_{i\neq j}K_{h_{2}}\left(\eta_{i}-\eta_{j}\right)/\sum_{i\neq j}K_{h_{2}}\left(\eta_{i}-\eta_{j}\right)e_{i}^{2},$$

$$\hat{g}\left(Z_{i}^{⊤}\gamma \right)=\sum_{i\neq j}K_{h_{1}}\left(Z_{i}^{⊤}\gamma -Z_{j}^{⊤}\gamma \right)\left(Y_{i}-X_{i}^{⊤}\theta \right)/\sum_{i\neq j}K_{h_{1}}\left(Z_{i}^{⊤}\gamma -Z_{j}^{⊤}\gamma \right)$$

and

$$\begin{array}{ccc} {\hat{g}}^{\prime }\left(Z_{i}^{⊤}\gamma \right) & = & h_{1}^{-1}\left\{\sum_{i\neq j}K_{h_{1}}^{\prime }\left(Z_{i}^{⊤}\gamma -Z_{j}^{⊤}\gamma \right)\left(Y_{i}-X_{i}^{⊤}\theta \right)\sum_{i\neq j}K_{h_{1}}\left(Z_{i}^{⊤}\gamma -Z_{j}^{⊤}\gamma \right)\right\}\\ & - & \sum_{i\neq j}K_{h_{1}}\left(Z_{i}^{⊤}\gamma -Z_{j}^{⊤}\gamma \right)\left(Y_{i}-X_{i}^{⊤}\theta \right)\\ & \times & \sum_{i\neq j}K_{h_{1}}^{\prime }\left(Z_{i}^{⊤}\gamma -Z_{j}^{⊤}\gamma \right)\}/{\left\{\sum_{i\neq j}K_{h_{1}}\left(Z_{i}^{⊤}\gamma -Z_{j}^{⊤}\gamma \right)\right\}}^{2}. \end{array}$$

We propose the following estimating equations for PLSIM:

(2) $$\begin{array}{c} \left\{\begin{array}{c} \frac{1}{\sqrt{n}}\sum_{i=1}^{n}{\tilde{\varepsilon }}_{i}\hat{w}\left(X_{i},Z_{i}\right)\left[X_{i}-\frac{\hat{E}\left\{\hat{w}\left(X,Z\right)X|Z_{i}^{⊤}\gamma \right\}}{\hat{E}\left\{\hat{w}\left(X,Z\right)|Z_{i}^{⊤}\gamma \right\}}\right]=0,\\ \frac{1}{\sqrt{n}}\sum_{i=1}^{n}{\tilde{\varepsilon }}_{i}\hat{w}\left(X_{i},Z_{i}\right){\hat{g}}^{\prime }\left(Z_{i}^{⊤}\gamma \right)\left[Z_{-1,i}-\frac{\hat{E}\left\{\hat{w}\left(X,Z\right)Z_{-1}|Z_{i}^{⊤}\gamma \right\}}{\hat{E}\left\{\hat{w}\left(X,Z\right)|Z_{i}^{⊤}\gamma \right\}}\right]=0, \end{array}\right. \end{array}$$

where ${\hat{w}}_{i}\equiv \hat{w}\left(X_{i},Z_{i}\right)$ and ${\tilde{\varepsilon }}_{i}=Y_{i}-\theta^{⊤}X_{i}-\hat{g}\left(Z_{i}^{⊤}\gamma \right)$ denotes the residual term for i=1,⋯,n.

Attempting to estimate var(ε∣X,Z) or w(X,Z) by applying nonparametric regression to the residuals and the covariates (X, Z) poses a significant challenge, as this constitutes a high-dimensional problem that is highly susceptible to the curse of dimensionality. To simplify the estimation of w(X,Z), we assume there exists a function $\eta_{i}=\eta \left(X_{i},Z_{i}\right)$ satisfying $\mathrm{var}\left(\varepsilon_{i}|X_{i},Z_{i}\right)=\mathrm{var}\left(\varepsilon_{i}|\eta_{i}\right)$, where $\eta_{i}$ is a known low-dimensional function of the covariates $\left(X_{i},Z_{i}\right)$, i=1,⋯,n. For instance, η could take the form $\theta^{⊤}X$, implying that the error variance depends solely on a linear combination of *X*. Alternatively, η could be $\gamma^{⊤}Z$, indicating dependence only on *Z*, or it could represent a combination of both or other structures. A similar assumption is also present in Ma and Zhu [6]. In practice, a reasonable approximation of η can be obtained using standard procedures for modeling heteroscedasticity, based on residuals from a preliminary model fit. It should be noted, however, that this assumption can be relaxed to incorporate intermediate multivariate frameworks, such as additive structures, thereby preserving univariate convergence rates while maintaining considerable flexibility in variance modeling.

Define

(3) $$S_{eff}=w\varepsilon {\left(X^{⊤}-\frac{E(wX^{⊤}|Z^{⊤}\gamma )}{E(w|Z^{⊤}\gamma )},\left\{Z_{-1}^{⊤}-\frac{E(wZ_{-1}^{⊤}|Z^{⊤}\gamma )}{E(w|Z^{⊤}\gamma )}\right\}g^{\prime }\left(Z^{⊤}\gamma \right)\right)}^{⊤}.$$

According to Ma and Zhu [6], $S_{eff}$ is the semiparametric efficient score, and the estimator ${\left({\hat{\theta }}^{⊤},{\hat{\gamma }}^{⊤}\right)}^{⊤}$, based on (2), is doubly robust and efficient with fixed *p* and *r*. Double robustness means that a consistent estimator of the target parameter can be obtained as long as one of the two models is correctly specified. For example, we can use an estimator $\hat{g}\left(\cdot \right)$ that is inconsistent for g(·); as long as the conditional expectation is consistently estimated, i.e., $\hat{E}\left(\cdot |Z^{⊤}\gamma \right)$ converges to $E(\cdot |Z^{⊤}\gamma )$, then expression (3) will yield consistent estimators for θ and γ. The converse also holds: if $\hat{g}\left(\cdot \right)$ is consistent for g(·), then consistency of the estimators in (3) is maintained even if the model for the conditional expectation is misspecified. However, the doubly robust and efficient property of the estimator achieved by solving (2) is no longer valid when *p* and *r* tend to infinity as n→∞.

Our goal is, under high-dimensional sparse setting, to develop new estimation and variable selection approaches for heteroscedastic model (1) by using the PEL method. In order to construct the PEL function, we need to propose an auxiliary random vector using $S_{eff}$. Define

$$\xi_{i}\left(\theta ,\gamma \right)=w_{i}\varepsilon_{i}{\left(X_{i}^{⊤}-\frac{E(w_{i}X_{i}^{⊤}|Z_{i}^{T}\gamma )}{E(w_{i}|Z_{i}^{⊤}\gamma )},\left\{Z_{-1,i}^{⊤}-\frac{E(w_{i}Z_{-1,i}^{⊤}|Z_{i}^{⊤}\gamma )}{E(w_{i}|Z_{i}^{⊤}\gamma )}\right\}g^{\prime }\left(Z_{i}^{⊤}\gamma \right)\right)}^{⊤}.$$

We have $E\{\xi_{i}\left(\theta ,\gamma \right)\}=0$ for i=1,⋯,n. Let $q=(q_{1}\cdots q_{n})$ satisfying $\sum_{i=1}^{n}q_{i}=1$, $q_{i}\geq 0$. For ${\left(\theta^{⊤},\gamma^{⊤}\right)}^{⊤}$, the EL function is written as

(4) $$L\left(\theta ,\gamma \right)=\sup\{\prod_{i=1}^{n}\left(nq_{i}\right):\sum_{i=1}^{n}q_{i}=1,q_{i}\geq 0,\sum_{i=1}^{n}q_{i}\xi_{i}\left(\theta ,\gamma \right)=0\}.$$

Since L(θ,γ) contains unknown functions, it cannot be directly used for statistical inference on ${\left(\theta^{⊤},\gamma^{⊤}\right)}^{⊤}$. A natural approach to solving this issue is to substitute the unknown functions in L(θ,γ) with their corresponding estimator provided above. For ${\left(\theta^{⊤},\gamma^{⊤}\right)}^{⊤}$, redefine the estimated EL function as

(5) $$\tilde{L}\left(\theta ,\gamma \right)=\sup\left\{\prod_{i=1}^{n}\left(nq_{i}\right):\sum_{i=1}^{n}q_{i}=1,q_{i}\geq 0,\sum_{i=1}^{n}q_{i}{\hat{\xi }}_{i}\left(\theta ,\gamma \right)=0\right\},$$

where ${\tilde{\varepsilon }}_{i}=Y_{i}-\theta^{⊤}X_{i}-\hat{g}\left(Z_{i}^{⊤}\gamma \right)$ and

$${\hat{\xi }}_{i}\left(\theta ,\gamma \right)={\hat{w}}_{i}{\tilde{\varepsilon }}_{i}{\left(X_{i}^{⊤}-\frac{\hat{E}\left({\hat{w}}_{i}X_{i}^{⊤}|Z_{i}^{⊤}\gamma \right)}{\hat{E}\left({\hat{w}}_{i}|Z_{i}^{⊤}\gamma \right)},\left\{Z_{-1,i}^{⊤}-\frac{\hat{E}\left({\hat{w}}_{i}Z_{-1,i}^{⊤}|Z_{i}^{⊤}\gamma \right)}{\hat{E}\left({\hat{w}}_{i}|Z_{i}^{⊤}\gamma \right)}\right\}{\hat{g}}^{\prime }\left(Z_{i}^{⊤}\gamma \right)\right)}^{⊤}.$$

Define the PEL estimator ${\left({\hat{\theta }}^{⊤},{\hat{\gamma }}^{⊤}\right)}^{⊤}$ as the maximizer of

(6) $$\log\left\{\tilde{L}\left(\theta ,\gamma \right)\right\}-n\sum_{i=1}^{p}p_{\tau }\left(\right|\theta_{i}\left|\right)-n\sum_{i=1}^{r}p_{\nu }\left(\right|\gamma_{i}\left|\right),$$

where $p_{\tau }\left(t\right)$ and $p_{\nu }\left(t\right)$ are the penalty functions with tuning parameters τ and ν, respectively.

Many commonly used penalty functions have been studied, i.e., the $L_{1}$ penalty (Donoho and Johnstone [26]); $L_{2}$ penalty (Hoerl and Kennard [27]); LASSO penalty (Tibshirani [11]); and SCAD penalty (Fan and Li [12]). It is well known that the SCAD penalty has the oracle property. Therefore, in this article, we consider PEL for a heteroscedastic PLSIM by using the SCAD penalty. Its first derivative satisfies

$$p_{\nu }^{\prime }\left(t\right)=\nu \left\{\frac{{\left(a\nu -t\right)}_{+}}{(a-1)\nu }I\left(t>\nu \right)+I\left(t\leq \nu \right)\right\},$$

where I(·) denotes an indicator function and *a* is a constant with a>2.

Combining the Lagrange multiplier method and Equation (5), we have

(7) $$q_{i}=\frac{1}{n}\frac{1}{1+\lambda^{⊤}{\hat{\xi }}_{i}\left(\theta ,\gamma \right)},$$

and λ satisfies

(8) $$\frac{1}{n}\sum_{i=1}^{n}\frac{{\hat{\xi }}_{i}\left(\theta ,\gamma \right)}{1+\lambda^{⊤}{\hat{\xi }}_{i}\left(\theta ,\gamma \right)}=0.$$

By substituting Equation (7) into $\tilde{L}\left(\theta ,\gamma \right)$, we can show that maximizing (6) corresponds to minimizing

(9) $${\tilde{\ell }}_{p}\left(\theta ,\gamma \right)=2\sum_{i=1}^{n}\log\left\{1+\lambda^{⊤}{\hat{\xi }}_{i}\left(\theta ,\gamma \right)\right\}+n\sum_{i=1}^{p}p_{\tau }\left(\right|\theta_{i}\left|\right)+n\sum_{i=1}^{r}p_{\nu }\left(\right|\gamma_{i}\left|\right).$$

Therefore, ${\left({\hat{\theta }}^{T},{\hat{\gamma }}^{⊤}\right)}^{⊤}$ can also be defined to be the minimization of (9).

Let $A_{1}=\left\{j:\theta_{0j}\neq 0\right\}$ and $A_{2}=\left\{j:\gamma_{0j}\neq 0\right\}$, and denote the cardinalities of $A_{1}$ and $A_{2}$ as $d_{1}$ and $d_{2}$, where $\theta_{0}$ and $\gamma_{0}$ are the true values of θ and γ respectively. Without loss of generality, we write $\theta ={\left(\theta_{1}^{⊤},\theta_{2}^{⊤}\right)}^{⊤}$, where $\theta_{1}\in R^{d_{1}}$ and $\theta_{2}\in R^{p-d_{1}}$ represent θ’s nonzero and zero components, respectively. $\gamma ={\left(\gamma_{1}^{⊤},\gamma_{2}^{⊤}\right)}^{⊤}$ can be similarly partitioned, where $\gamma_{1}\in R^{d_{2}}$ and $\gamma_{2}\in R^{r-d_{2}}$. Analogously, the true parameter values $\theta_{0}$ and $\gamma_{0}$ can be decomposed as $\theta_{0}={\left(\theta_{10}^{⊤},0\right)}^{⊤}$ and $\gamma_{0}={\left(\gamma_{10}^{⊤},0\right)}^{T}$. For notational purposes, let $I_{p}={\left(H_{1}^{⊤},H_{2}^{⊤}\right)}^{⊤}$ and $I_{r-1}={\left(H_{3}^{⊤},H_{4}^{⊤}\right)}^{⊤}$, where $H_{1}\in R^{d_{1}\times p}$, $H_{2}\in R^{(p-d_{1})\times p}$, $H_{3}\in R^{d_{2}\times \left(r-1\right)}$, and $H_{4}\in R^{\left(r-1-d_{2}\right)\times \left(r-1\right)}$.

To derive asymptotic properties of the proposed PEL estimator, the following conditions are necessary.

*Condition 1*. $K_{h}\left(\cdot \right)$ is symmetric with $K_{h}^{\prime }\left(\cdot \right)$ continuous on [−1,1].

*Condition 2*. The bandwidth $h_{i}$, for i=1,2,3, satisfies the following asymptotic assumption: (1) $nh_{1}^{8}\to 0$, $nh_{1}^{4}\to \infty$, $h_{2}=O\left(1/\sqrt[{5}]{n}\right)$ and $h_{3}=O\left(1/\sqrt[{5}]{n}\right)$; (2) $\log^{2}\left(n\right)/\left(nh_{i}\right)\to 0$, $\log^{4}\left(n\right)/\left(nh_{1}h_{i}\right)\to 0$ and $h_{1}^{4}\log^{2}\left(n\right)/h_{i}\to 0$.

*Condition 3*. Let $\mathrm{Var}\left(X_{i}\right)=\Sigma_{xi}$ and $\mathrm{Var}\left(Z_{i}\right)=\Sigma_{zi}$, i=1⋯n. For $\Sigma_{xi}$ and $\Sigma_{zi}$, their eigenvalues satisfy $C_{1}\leq \Gamma_{1}\left(\Sigma_{xi}\right)\leq \cdots \leq \Gamma_{p}\left(\Sigma_{xi}\right)\leq C_{2}$ and $C_{1}\leq \Gamma_{1}\left(\Sigma_{zi}\right)\leq \cdots \leq \Gamma_{r}\left(\Sigma_{zi}\right)\leq C_{2}$ for some constants $0<C_{1}<C_{2}$, i=1⋯n. In addition, $E(\varepsilon^{4+\delta }|X,Z)<\infty$, where δ is a positive constant.

*Condition 4*. Let v(·) and η=η(X,Z) satisfy $E\left(\varepsilon^{2}|X,Z\right)=v\left(\eta \right)$, where $0<C_{1}<v\left(\cdot \right)<C_{2}<\infty$, and $C_{1}$ and $C_{2}$ are positive constants. Moreover, $\mathrm{Var}(X_{i}|\eta \left(X_{i},Z_{i}\right))$ is positive definite with a bounded spectrum.

*Condition 5*. There exist $v_{1}\left(X,Z\right)$ values satisfying

$$\frac{\partial^{2}E\left(X|Z^{⊤}\gamma \right)}{\partial \gamma_{i}\partial \gamma_{j}},\frac{\partial^{2}E\left(Z|Z^{⊤}\gamma \right)}{\partial \gamma_{i}\partial \gamma_{j}},\frac{\partial^{2}E\left(w|Z^{⊤}\gamma \right)}{\partial \gamma_{i}\partial \gamma_{j}},\frac{\partial^{2}E\left(wZ|Z^{⊤}\gamma \right)}{\partial \gamma_{i}\partial \gamma_{j}},$$

$$\frac{\partial^{2}E\left(wX|Z^{⊤}\gamma \right)}{\partial \gamma_{i}\partial \gamma_{j}}<v_{1}\left(X,Z\right),Ev_{1}^{2}<\infty ,\left(i,j=1,\cdots ,p\right).$$

There also exist $v_{2}\left(X,Z\right)$ values such that

$$\frac{\partial^{3}\eta \left(X,Z\right)}{\partial \pi_{i}\partial \pi_{j}\partial \pi_{k}}<v_{2}\left(X,Z\right),Ev_{2}^{2}<\infty ,$$

where ${\left(X^{⊤},Z^{⊤}\right)}^{⊤}={\left(\pi_{1}\cdots \pi_{p+r}\right)}^{⊤}$ and i,j,k=1,⋯,p+r. There exist $v_{3}\left(X,Z\right)$ values satisfying

$$\frac{\partial^{4}g\left(Z^{⊤}\gamma \right)}{\partial \gamma_{i}\partial \gamma_{j}\partial \gamma_{k}\partial \gamma_{l}},\frac{\partial^{4}v\left(\eta \right)}{\partial \eta_{i_{1}}\partial \eta_{j_{1}}\partial \eta_{k_{1}}\partial \eta_{l_{1}}}<v_{3}\left(X,Z\right),Ev_{3}^{2}<\infty ,$$

where $\eta \in R^{p_{1}}$, i,j,k,l=1,⋯,p and $i_{1},j_{1},k_{1},l_{1}=1\cdots p_{1}$.

*Condition 6*. Assume η and $Z^{⊤}\gamma$ possess densities, denoted by $f_{\eta }\left(\eta \right)$ and $f_{Z^{⊤}\gamma }\left(Z^{⊤}\gamma \right)$, respectively, which are bounded away from zero and infinity. There exist $v_{4}\left(X,Z\right)$ values such that

$$\frac{\partial^{2}f_{Z^{⊤}\gamma }\left(Z^{⊤}\gamma \right))}{\partial \gamma_{i}\partial \gamma_{j}},\frac{\partial^{2}f_{\eta }\left(\eta \right)}{\partial \eta_{k}\partial \eta_{l}}<v_{4}\left(X,Z\right),Ev_{4}^{2}<\infty ,\left(i,j=1\cdots p;\;k,l=1\cdots p_{1}\right).$$

*Condition 7*. As n→∞, we assume p→∞ and $p/\sqrt[{5}]{n}\to 0$, and r→∞ and $r/\sqrt[{5}]{n}\to 0$.

*Condition 8*. All random elements *X*, ε, εX, and εZ have finite fourth moments.

*Condition 9*. Define

$$\xi_{n}\left(\theta ,\gamma \right)=w\varepsilon {\left(X^{⊤}-\frac{E(wX^{⊤}|Z^{⊤}\gamma )}{E(w|Z_{i}^{⊤}\gamma )},\left\{Z^{⊤}-\frac{E(wZ^{⊤}|Z^{⊤}\gamma )}{E(w|Z^{⊤}\gamma )}\right\}g^{\prime }\left(Z^{⊤}\gamma \right)\right)}^{⊤}.$$

As n→∞, we assume the following moments are uniformly bounded by a positive constant *C*: $E(∥\xi_{n}\left(\theta ,\gamma \right)/\sqrt{p}{∥}^{4})<C$, $E(∥ZX^{⊤}{∥}^{4})<C$, $E(∥XX^{⊤}{∥}^{4})<C$. Furthermore, we assume $E(∥XZ^{⊤}{∥}^{4})<\infty$.

*Condition 10*. As n→∞, $\tau {\left(p/n\right)}^{\frac{1}{2}}\to \infty$, $\nu {\left(r/n\right)}^{1/2}\to \infty$, $\min_{j\in A_{1}}\theta_{0j}/\tau \to \infty$, and $\min_{j\in A_{2}}\gamma_{0j}/\nu \to \infty$.

*Condition 11*. Assume $\max_{j\in A_{1}}P_{\tau }^{\prime }\left(\right|\theta_{0j}\left|\right)=o\left\{{\left(np\right)}^{-1/2}\right\}$, $\max_{j\in A_{2}}P_{\nu }^{\prime }\left(\right|\gamma_{0j}\left|\right)=o\left\{{\left(nr\right)}^{-1/2}\right\}$, $\max_{j\in A_{1}}P_{\tau }^{\prime \prime }\left(\right|\theta_{0j}\left|\right)=o\left\{{\left(p\right)}^{-1/2}\right\}$, and $\max_{j\in A_{2}}P_{\nu }^{\prime \prime }\left(\right|\gamma_{0j}\left|\right)=o\left\{{\left(r\right)}^{-1/2}\right\}$.

Conditions 1–6 support the existence of estimators ${\left({\hat{\theta }}^{T},{\hat{\gamma }}^{⊤}\right)}^{⊤}$. These conditions also ensure that the functions w(X,Z), $g(Z^{⊤}\gamma )$, and $g^{\prime }\left(Z^{⊤}\gamma \right)$ and the conditional expectations $E\{\hat{w}\left(X,Z\right)Z_{-1,i}|Z_{i}^{⊤}\gamma \}$, $E\{\hat{w}\left(X,Z\right)X|Z_{i}^{⊤}\gamma \}$, and $E\{\hat{w}\left(X,Z\right)|Z_{i}^{⊤}\gamma \}$ can be estimated with maintained precision. Moreover, these conditions also guarantee that nonparametric estimation does not alter the asymptotic behavior of the empirical likelihood ratio. As a result, the estimated PEL ratio $\tilde{L}\left(\theta ,\gamma \right)$ converges to the same asymptotic distribution as the standard PEL ratio L(θ,γ). Conditions 1–6 were also used by Ma and Zhu [6] as sufficient conditions for the double-robustness property of the estimators. Condition 7 serves as a technical requirement. Since determining the minimum upper bound is quite challenging, this condition is necessary, and the resulting bounds in the stochastic analysis are conservative. Condition 8 guarantees that the asymptotic variance exists for the estimator of the increasing-dimensional parameters ${\left(\theta^{T},\gamma^{⊤}\right)}^{⊤}$. Condition 9 restricts the tail probability behavior of the estimating equation. Condition 10 requires that the weakest signal remains stronger than the penalty parameter, and Condition 11 helps limit the influence of the penalty on the nonzero components. Conditions 10 and 11 are satisfied by a range of penalty functions, including those discussed in Fan and Li [12]. Due to the considerable theoretical challenges in establishing asymptotic properties for PEL methods in the context of diverging covariate dimensions, these conditions are intentionally stringent rather than minimally sufficient, and the resulting stochastic bounds are conservative.

In the following theorem, we will show the theoretical properties of the PEL estimator ${\left({\hat{\theta }}^{⊤},{\hat{\gamma }}^{⊤}\right)}^{⊤}$.

**Theorem** **1.** *As n→∞, under Conditions 1–11, we have*

*(1)*  *${\hat{\theta }}_{2}=0$ and ${\hat{\gamma }}_{2}=0$, with probability tending to 1;*
*(2)*  *$\sqrt{n}BI_{B}^{-1/2}\left\{{\left({\hat{\theta }}_{1}^{⊤},{\hat{\gamma }}_{1}^{⊤}\right)}^{⊤}-{\left(\theta_{10}^{⊤},\gamma_{10}^{⊤}\right)}^{⊤}\right\}\overset{L}{\to }N\left(0,G\right)$, where $B\in R^{\left(q_{1}+q_{2}\right)\times \left(p+r-1\right)}$, $BB^{⊤}\to G$ and G is a $\left(q_{1}+q_{2}\right)\times \left(q_{1}+q_{2}\right)$ matrix with fixed $q_{1}$ and $q_{2}$,*

$$\begin{array}{c} H_{0}=\left(\begin{array}{cc} H_{1} & 0\\ 0 & H_{3} \end{array}\right),\;H=\left(\begin{array}{cc} H_{2} & 0\\ 0 & H_{4} \end{array}\right), \end{array}$$

$$\begin{array}{c} B=\left(\begin{array}{cc} B_{1} & 0\\ 0 & B_{2} \end{array}\right),\;\;\;V=\left(\begin{array}{cc} V_{11} & V_{12}\\ V_{21} & V_{22} \end{array}\right),\;U=\left(\begin{array}{cc} U_{11} & U_{12}\\ U_{21} & U_{22} \end{array}\right) \end{array}$$

$$V_{11}=E\left\{wXX^{T}-\frac{E\left(wX|Z^{T}\gamma \right)E\left(wX^{T}|Z^{T}\gamma \right)}{E(w|Z^{T}\gamma )}\right\},$$

$$V_{12}=E\left[g^{\prime }\left(Z^{T}\gamma \right)\left\{wXZ_{-1}^{T}-\frac{E\left(wX|Z^{T}\gamma \right)E\left(wZ_{-1}^{T}|Z^{T}\gamma \right)}{E(w|Z^{T}\gamma )}\right\}\right],$$

$$V_{21}=E\left[g^{\prime }\left(Z^{T}\gamma \right)\left\{wZ_{-1}X^{T}-\frac{E\left(wZ_{-1}|Z^{T}\gamma \right)E\left(wX^{T}|Z^{T}\gamma \right)}{E(w|Z^{T}\gamma )}\right\}\right],$$

$$V_{22}=E\left[g^{\prime }{\left(Z^{T}\gamma \right)}^{2}\left\{wZ_{-1}Z_{-1}^{T}-\frac{E\left(wZ_{-1}|Z^{T}\gamma \right)E\left(wZ_{-1}^{T}|Z^{T}\gamma \right)}{E(w|Z^{T}\gamma )}\right\}\right],$$

$$U_{11}=E\left\{\frac{\partial \xi_{i}\left(\theta ,\gamma \right)}{\partial \theta^{⊤}}V^{-1}\frac{\partial \xi_{i}\left(\theta ,\gamma \right)}{\partial \theta }\right\},\;\;U_{12}=E\left\{\frac{\partial \xi_{i}\left(\theta ,\gamma \right)}{\partial \theta^{⊤}}V^{-1}\frac{\partial \xi_{i}\left(\theta ,\gamma \right)}{\partial \gamma }\right\},$$

$$U_{21}=E\left\{\frac{\partial \xi_{i}\left(\theta ,\gamma \right)}{\partial \gamma^{⊤}}V^{-1}\frac{\partial \xi_{i}\left(\theta ,\gamma \right)}{\partial \theta }\right\},\;\;U_{12}=E\left\{\frac{\partial \xi_{i}\left(\theta ,\gamma \right)}{\partial \gamma^{⊤}}V^{-1}\frac{\partial \xi_{i}\left(\theta ,\gamma \right)}{\partial \gamma }\right\},$$

*$I_{B}=H_{0}U^{-1}VU^{-1}H_{0}^{⊤}-H_{0}U^{-1}H^{⊤}{\left(HU^{-1}H^{⊤}\right)}^{-1}H_{2}U^{-1}VA^{-1}H_{2}^{⊤}{\left(HU^{-1}H^{⊤}\right)}^{-1}HU^{-1}H_{0}^{⊤}$, and $\overset{L}{\to }$ denotes convergence in distribution.*

In Theorem 1, *B* projects the diverging dimensional parameter vector ${\left(\theta_{1}^{⊤},\gamma_{1}^{⊤}\right)}^{⊤}$ onto a fixed $\left(q_{1}+q_{2}\right)$-dimensional subspace.

**Remark** **1.** *Theorem 1 proves that the proposed estimator satisfies the oracle property. Specifically, the components of $\theta_{20}$ and $\gamma_{20}$ are estimated as zero, and the PEL estimator of the nonzero components $\theta_{10}$ and $\gamma_{10}$ is efficient, with probability tending to 1.*

Next, we will describe the construction of confidence regions and hypothesis testing for ${\left(\theta^{⊤},\gamma^{⊤}\right)}^{⊤}$ using the PEL method. Consider testing the following null and alternative hypotheses:

$$H_{0}:L_{n}{\left(\theta_{0}^{⊤},\gamma_{0}^{⊤}\right)}^{⊤}=0\;\;\;\;\mathrm{vs}.\;\;\;\;H_{1}:L_{n}{\left(\theta_{0}^{⊤},\gamma_{0}^{⊤}\right)}^{⊤}\neq 0,$$

where $L_{n}\in R^{\left(q_{1}+q_{2}\right)\times \left(p+r-1\right)}$ satisfies that, for the fixed $q_{1}$ and $q_{2}$,

$$\begin{array}{c} L_{n}=\left(\begin{array}{cc} L_{n1} & 0\\ 0 & L_{n2} \end{array}\right),L_{n}L_{n}^{⊤}=\left(\begin{array}{cc} I_{q_{1}} & 0\\ 0 & I_{q_{2}} \end{array}\right), \end{array}$$

$L_{n1}\in R^{q_{1}\times p}$, $L_{n2}\in R^{q_{2}\times r-1}$, and $I_{q_{1}}$ and $I_{q_{2}}$ are the $q_{1}$-dimensional and $q_{2}$-dimensional identity matrixes, respectively. Not only can we use this type of hypothesis to test the hypothesis for individual and multiple components of ${\left(\theta_{0}^{⊤},\gamma_{0}^{⊤}\right)}^{⊤}$, but we can also use it to test the hypotheses about linear functions of ${\left(\theta_{0}^{⊤},\gamma_{0}^{⊤}\right)}^{⊤}$.

Similar to the EL ratio for the PLSIM in [3], we can construct the PEL ratio statistic as

(10) $$\tilde{\ell }\left(L_{n}\right)=-\left\{{\tilde{\ell }}_{p}\left(\hat{\theta },\hat{\gamma }\right)-\min_{\left(\theta ,\gamma \right):L_{n}{\left(\theta^{⊤},\gamma^{⊤}\right)}^{⊤}=0}{\tilde{\ell }}_{p}\left(\theta ,\gamma \right)\right\}.$$

The following theorem shows the properties of the PEL ratio statistic for model (1).

**Theorem** **2.** *As n→∞, under the null hypothesis and Conditions 1–11, we have*

$$\tilde{\ell }\left(L_{n}\right)\overset{L}{\to }\chi_{q_{1}+q_{2}}^{2}.$$

The standard PEL ratio, under some regular conditions, converges in law to a chi-square distribution. This is one of the most important properties of the PEL method, and similar conclusions can be found in Fang et al. [7] and Tang and Leng [24], among others. Theorem 2 shows that, under Conditions 1–11, the estimated PEL ratio $\tilde{L}\left(\theta ,\gamma \right)$ converges to the same asymptotic distribution as the standard PEL ratio L(θ,γ). This result provides a convenient approach for testing hypotheses and constructing data-driven confidence regions without any shape constraints. Combined with the oracle property of the PEL method established in Theorem 1, these findings demonstrate the robustness and efficiency of the PEL method for PLSIMs.

Confidence regions for ${\left(\theta^{⊤},\gamma^{⊤}\right)}^{⊤}$ can be constructed using Theorem 2; that is,

(11) $$I_{\alpha }=\left\{{\left(\theta^{⊤},\gamma^{⊤}\right)}^{⊤}:-\left\{{\tilde{\ell }}_{p}\left(\hat{\theta },\hat{\gamma }\right)-\min_{\left(\theta ,\gamma \right):L_{n}{\left(\theta^{⊤},\gamma^{⊤}\right)}^{⊤}=0}{\tilde{\ell }}_{p}\left(\theta ,\gamma \right)\right\}\leq \chi_{q_{1}+q_{2},\left(1-\alpha \right)}^{2}\right\},$$

where $\chi_{q_{1}+q_{2},\left(1-\alpha \right)}^{2}$ is the 1−α quantile of the $\chi_{q_{1}+q_{2}}^{2}$ distribution with $q_{1}+q_{2}$ degrees of freedom. Here $I_{\alpha }$ provides an asymptotic confidence region for ${\left(\theta^{⊤},\gamma^{⊤}\right)}^{⊤}$ with confidence level 1−α, i.e., as n→∞, $P(L_{n}\left(\theta ,\gamma \right)\in I_{\alpha })\to 1-\alpha$.

**Remark** **2.** *In this article, we develop a PEL method for simultaneous variable selection and parameter estimation in high-dimensional sparse heteroscedastic PLSIMs, and this requires n>p and n>r. When this is violated in practice, one can first adopt SIS, proposed by Fan and Lv [28], to reduce the dimensionality to a moderate level below sample size.*

## 3. Penalized Empirical Likelihood for PLM and SIM

For two special cases of model (1), we develop PEL estimators for parameters θ and γ. If γ=1, model (1) reduces to a heteroscedastic PLM, and it can be written as

(12) $$Y_{i}=\theta^{⊤}X_{i}+g\left(Z_{i}\right)+\varepsilon_{i},\;for\;\;i=1,\cdots ,n.$$

Consider the PEL method for the high-dimensional PLM. Redefine the EL function for θ and ${\hat{\xi }}_{1i}\left(\theta \right)$ as

(13) $$\tilde{L}\left(\theta \right)=\sup\left\{\prod_{i=1}^{n}\left(nq_{i}\right):\sum_{i=1}^{n}q_{i}=1,q_{i}\geq 0,\sum_{i=1}^{n}q_{i}{\hat{\xi }}_{1i}\left(\theta \right)=0\right\},$$

and

(14) $${\hat{\xi }}_{1i}\left(\theta \right)={\hat{w}}_{i}\left\{Y_{i}-X_{i}^{T}\theta -\hat{g}\left(Z_{i}\right)\right\}\left\{X_{i}-\frac{\hat{E}\left({\hat{w}}_{i}X_{i}|Z_{i}\right)}{\hat{E}\left({\hat{w}}_{i}|Z_{i}\right)}\right\},i=1,\cdots ,n.$$

The PEL function for the model (12) can be written as

(15) $${\tilde{\ell }}_{p}\left(\theta \right)=2\sum_{i=1}^{n}\log\left\{1+\lambda^{⊤}{\hat{\xi }}_{1i}\left(\theta \right)\right\}+n\sum_{i=1}^{p}p_{\tau }\left(\right|\theta_{i}\left|\right).$$

We state the similar results as follows.

**Corollary** **1.** *As n→∞, under Conditions 1–11, we have*

*(1)*  *${\hat{\theta }}_{2}=0$, with probability tending to 1;*
*(2)*  *$\sqrt{n}B_{1}I^{-1/2}\{\left({\hat{\theta }}_{1}-\left(\theta_{10}\right)\right\}\overset{L}{\to }N\left(0,G\right)$, where $B_{1}\in R^{q_{1}\times p}$, $B_{1}B_{1}^{⊤}\to G_{1}$, $G_{1}$ is a $q_{1}\times q_{1}$ matrix with fixed $q_{1}$ and $\overset{L}{\to }$ stands for convergence in distribution.*

Consider testing the following null and alternative hypotheses:

$$H_{0}:L_{n1}\theta_{0}=0\;\;\;\;\mathrm{vs}.\;\;\;\;H_{1}:L_{n1}\theta_{0}\neq 0,$$

where $L_{n1}\in R^{q_{1}\times p}$ satisfies that, for the fixed $q_{1}$, $L_{n1}L_{n1}^{⊤}=I_{q_{1}}$, where $I_{q_{1}}$ is a $q_{1}$-dimensional identity matrix. The PEL ratio statistic can be constructed as follows:

(16) $$\tilde{\ell }\left(L_{n1}\right)=-\left\{{\tilde{\ell }}_{p}\left(\hat{\theta }\right)-\min_{\theta :L_{n1}\theta =0}{\tilde{\ell }}_{p}\left(\theta \right)\right\}.$$

**Corollary** **2.** *As n→∞, under the null hypothesis and Conditions 1–11, we have*

$$\tilde{\ell }\left(L_{n1}\right)\overset{L}{\to }\chi_{q_{1}}^{2}.$$

Next, we consider the following SIM with a diverging number of parameters, which is another special case of model (1).

(17) $$Y_{i}=g\left(Z_{i}^{⊤}\gamma \right)+\varepsilon_{i},\;\;\;E\left(\varepsilon_{i}|X_{i},Z_{i}\right)=0\;\;\;i=1,\cdots ,n.$$

Redefine ${\hat{\xi }}_{2i}\left(\gamma \right)$ as

$${\hat{\xi }}_{2i}\left(\gamma \right)={\hat{w}}_{i}\left(Y_{i}-\hat{g}\left(Z_{i}^{⊤}\gamma \right)\right)\left\{Z_{-1,i}-\frac{\hat{E}\left({\hat{w}}_{i}Z_{-1,i}|Z_{i}^{⊤}\gamma \right)}{\hat{E}\left({\hat{w}}_{i}|Z_{i}^{⊤}\gamma \right)}\right\}{\hat{g}}^{\prime }\left(Z_{i}^{⊤}\gamma \right),i=1,\cdots ,n,$$

and rewrite the PEL ratio (9) as

(18) $${\tilde{\ell }}_{p}\left(\gamma \right)=2\sum_{i=1}^{n}\log\left\{1+\lambda^{⊤}{\hat{\xi }}_{2i}\left(\gamma \right)\right\}+n\sum_{i=1}^{r}p_{\nu }\left(\right|\gamma_{i}\left|\right).$$

**Corollary** **3.** *As n→∞, nnder Conditions 1–11, we have*

*(1)*  *${\hat{\gamma }}_{2}=0$, with probability tending to 1;*
*(2)*  *$\sqrt{n}B_{2}I^{-1/2}\{\left({\hat{\gamma }}_{1}-\left(\gamma_{10}\right)\right\}\overset{L}{\to }N\left(0,G\right)$, where $B_{2}\in R^{q_{2}\times r-1}$, $B_{2}B_{2}^{⊤}\to G_{2}$, and $G_{2}$ is a $q_{2}\times q_{2}$ matrix with fixed $q_{2}$.*

Consider testing the following null and alternative hypotheses:

$$H_{0}:L_{n2}\gamma_{0}=0\;\;\;\;\mathrm{vs}.\;\;\;\;H_{1}:L_{n2}\gamma_{0}\neq 0,$$

where $L_{n2}\in R^{q_{2}\times r}$ satisfies that, for the fixed $q_{2}$, $L_{n2}L_{n2}^{⊤}=I_{q_{2}}$ and $I_{q_{2}}$ is a $q_{2}$-dimensional identity matrix. The PEL ratio statistic for γ can be written as follows:

(19) $$\tilde{\ell }\left(L_{n2}\right)=-\left\{{\tilde{\ell }}_{p}\left(\hat{\gamma }\right)-\min_{\gamma :L_{n2}\gamma =0}{\tilde{\ell }}_{p}\left(\gamma \right)\right\}.$$

**Corollary** **4.** *As n→∞, under the null hypothesis and Conditions 1–11, we have*

$$\tilde{\ell }\left(L_{n2}\right)\overset{L}{\to }\chi_{q_{2}}^{2}.$$

## 4. Simulations

First, we describe how to solve the optimization problems introduced by the PEL. The minimizer of the PEL ratio is obtained through the local quadratic approximation algorithm. For the PEL estimator, we minimize (9) using a nested optimization algorithm. The following steps outline the nested algorithm for calculating the PEL estimator by minimizing Equation (9).

Step 1: We use the estimation procedure (a relatively simple but inefficient estimation method) described in Section 2 of Ma and Zhu [6] to obtain an initial estimator ${\left(\theta^{0⊤},\gamma^{0⊤}\right)}^{⊤}$.
Step 2: Obtain $\hat{g}\left(Z_{i}^{⊤}\gamma \right)$, ${\hat{g}}^{\prime }\left(Z_{i}^{⊤}\gamma \right)$, $\hat{w}\left(X_{i},Z_{i}\right)$, $\hat{E}\left\{\hat{w}\left(X,Z\right)|Z_{i}^{⊤}\gamma \right\}$, $\hat{E}\left\{\hat{w}\left(X,Z\right)X|Z_{i}^{⊤}\gamma \right\}$, and $\hat{E}\left\{\hat{w}\left(X,Z\right)Z_{-1}|Z_{i}^{⊤}\gamma \right\}$ described above using fixed values of ${\left(\theta^{⊤},\gamma^{⊤}\right)}^{⊤}$.
Step 3: Obtain the auxiliary random vector ${\hat{\xi }}_{i}\left(\theta ,\gamma \right)$.
Step 4: Use Newton’s method to minimize (9) with respect to λ for fixed values of ${\left(\theta^{⊤},\gamma^{⊤}\right)}^{⊤}$.
Step 5: Use the local quadratic approximation algorithm to minimize (9) with respect to ${\left(\theta^{⊤},\gamma^{⊤}\right)}^{⊤}$ for fixed values of λ obtained from Step 4.
Step 6: Iterate Steps 4 and 5 until ${\left(\theta^{⊤},\gamma^{⊤}\right)}^{⊤}$ converges.

Assume that ${\left(\theta^{0⊤},\gamma^{0⊤}\right)}^{⊤}$ is an initial value of ${\left(\theta^{⊤},\gamma^{⊤}\right)}^{⊤}$, and $\theta_{j}^{\left(k\right)}$ and $\gamma_{l}^{\left(k\right)}$ are the *k*-th step estimators of $\theta_{j}$ and $\gamma_{l}$, respectively. When $\theta_{j}^{\left(k\right)}$ ($|\theta_{j}^{\left(k\right)}|<\varsigma$) or $\gamma_{l}^{\left(k\right)}$ (where $|\gamma_{l}^{\left(k\right)}|\;<\varsigma$) are very close to 0, we set ${\hat{\theta }}_{j}^{\left(k\right)}=0$ or ${\hat{\gamma }}_{l}^{\left(k\right)}=0$, where ς is a predefined small positive tolerance. If $\theta_{j}^{\left(k\right)}\neq 0$, $p_{\tau }\left(\right|\theta_{j}\left|\right)$ can be locally approximated by $p_{\tau }\left(\right|\theta_{j}^{\left(k\right)}\left|\right)+\frac{1}{2}\{p_{\tau }^{\prime }\left(\right|\theta_{j}^{\left(k\right)}\left|)/\right|\theta_{j}^{\left(k\right)}\left|\right\}\left\{\theta_{j}^{2}-{\left(\theta_{j}^{\left(k\right)}\right)}^{2}\right\}$. Similarly, we can use $p_{\nu }\left(\right|\gamma_{l}^{\left(k\right)}\left|\right)+\frac{1}{2}\{p_{\nu }^{\prime }\left(\right|\gamma_{l}^{\left(k\right)}\left|)/\right|\gamma_{l}^{\left(k\right)}\left|\right\}\left\{\gamma_{l}^{2}-{\left(\gamma_{l}^{\left(k\right)}\right)}^{2}\right\}$ to approximate $p_{\nu }\left(\right|\gamma_{l}\left|\right)$ when $\gamma_{l}^{\left(k\right)}\neq 0$. These procedures are repeated until $∥{\left(\theta^{(j+1)⊤},\gamma^{(j+1)⊤}\right)}^{⊤}-{\left(\theta^{j⊤},\gamma^{j⊤}\right)}^{⊤}∥\;<\varsigma_{1}$, where $\varsigma_{1}$ is a very small positive number.

Next, we present simulation studies to illustrate the properties of the PEL inference for a heteroscedastic PLSIM.

**Example** **1.** *For the PLSIM, we generated $X_{1}$ from a Poisson distribution with parameter 2, $X_{p}$ from a binomial distribution with a success probability 0.6, $X_{j}$ from the uniform distribution U(0,1) for j=2,⋯,(p−1), and $Z_{k}$ from the normal distribution with mean 0 and variance 1. Using ${\left(X^{⊤},Z^{⊤}\right)}^{⊤}$, we generated responses from $Y∼N(X^{T}\theta +\exp\left(Z^{T}\gamma \right),\mathrm{Var}\left(Y\right)=|Z^{T}\gamma |)$. Let $\theta ={\left(2,\cdots ,1,0\right)}^{⊤}$ and $\gamma ={\left(1,1.5,-2,\cdots ,0\right)}^{⊤}$. We consider dimensions p=10,20 and r=10,20, and sample sizes n=50,100, and 200, respectively. We applied the cross-validation method to select the penalty parameters τ and ν. In order to compare the influence of the kernel function, we consider using the cosine kernel, defined as $\frac{\pi }{4}\cos\left(\frac{\pi t}{2}\right)\cdot I\left(t\leq 1\right)$, and the Epanechnikov kernel, given by $K\left(t\right)=\frac{3}{4}{\left(1-t^{2}\right)}_{+}$, respectively. In accordance with Condition 2, the bandwidth was set to $n^{-1/5}$, resulting in h≈0.45 at n=50, h≈0.4 at n=100, and h≈0.35 at n=200. Furthermore, to examine the robustness of the bandwidth selection, a grid search algorithm was also employed. For each of these settings, we repeated the simulation 500 times, and the results are reported in Table 1 and Table 2.*

*From Table 1 and Table 2, we observe that (1) for fixed p and n, as the sample size increases, the accuracy of variable selection improves and the standard deviation of the estimation decreases; (2) the choice of kernel function has a relatively minor influence on the results. Overall, the Epanechnikov kernel performs slightly better in estimation than the cosine kernel.*

**Example** **2.** *To consider the performance of the presented method for dependent covariates, we generated predictors by ${\left(X,Z\right)}^{T}∼N\left(0,\Sigma \right)$, where $\sigma_{ij}=0.3^{\left|i-j\right|}$, and generated responses by $Y∼N(X^{T}\theta +\exp\left(Z^{T}\gamma \right),\mathrm{Var}\left(Y\right)=|Z^{T}\gamma |)$. Let $\theta ={\left(1,\cdots ,-1,0\right)}^{⊤}$ and $\gamma ={\left(1,1,2,1,\cdots ,0\right)}^{⊤}$. We consider dimensions p=20,30 and r=20,30, and sample sizes of n=200,400, respectively. We applied the Epanechnikov kernel functions $K\left(t\right)=\frac{3}{4}{\left(1-t^{2}\right)}_{+}$, and applied the cross-validation method to select the penalty parameters τ and ν. According to Condition 2, the bandwidth was set to be $n^{-1/5}$, which means that h≈0.35 when n=200, and h≈0.3 when n=400. Because Lai et al. [16] also studied parameter estimation and variable selection for a heteroscedastic PLSIM, we computed their estimator (PVS) in this simulation study for the purpose of comparison. For each of these settings, we repeated the simulation 500 times, and the results are reported in Table 3 and Table 4.*

*From Table 3 and Table 4, it can be observed that (1) both estimators (PEL and PVS) yield estimates close to the true parameter values, with PEL exhibiting slightly smaller standard deviations than PVS; (2) in terms of variable selection, PEL produces, on average, fewer false zeros than PVS. Furthermore, the PEL method is a nonparametric methodology that retains the advantages of parametric likelihood while possessing double robustness. In contrast, the PVS method is a semiparametric efficient method and does not possess double robustness. For instance, when the model is misspecified, the performance of the PVS estimator is adversely affected. Thus, the proposed PEL method demonstrates favourable performance and outperforms the PVS method.*

## 5. Real Data Application

We will demonstrate the proposed methodology through application of a PLSIM to the AIDS Clinical Trials Group Protocol 175 (ACTG175) dataset (Hammer et al. [29]; https://www.nejm.org/doi/full/10.1056/NEJM199610103351501#tab-contributors (accessed on 5 August 2025)), previously examined by Lai and Wang [30]. The CD4 glycoprotein functions as an essential T-cell receptor (TCR) coreceptor that facilitates antigen-presenting cell interactions, establishing CD4 cell count as the primary immunological endpoint for comparing antiretroviral treatment effects during predefined observation periods in HIV clinical research. ACTG175 evaluates four distinct antiretroviral regimens: didanosine (ddI), zidovudine (ZDV) monotherapy, ZDV+ddI, and ZDV+zalcitabine, utilizing a balanced randomization design to assign 2138 eligible participants across therapeutic arms. The trial results demonstrate that structured antiretroviral interventions effectively reduce disease progression risks among clinically asymptomatic individuals with intermediate-stage HIV infection.

We aimed to construct a PLSIM to analyze subject responses under zidovudine (ZDV) monotherapy. Our analysis utilizes a curated subset of the ACTG175 cohort comprising 320 patients with complete CD4 endpoint data, derived from an initial pool of 521 subjects exhibiting baseline CD4 counts between 200–500 cells/mm^3^. The response variable *Y* (CD496) quantifies CD4 cell counts at 96±5 weeks post-treatment. Predictors include the following:

*Linear component*: Discrete covariates $x_{1}$ (drugs (history of IV drug use (0 = no, 1 = yes))), $x_{2}$ (str2 (antiretroviral history (0 = naive, 1 = experienced))), $x_{3}$ (gender (0 = F, 1 = M)), $x_{4}$ (symptom (symptomatic indicator (0 = asymp, 1 = symp))), $x_{5}$ (race (0 = White, 1 = non-white)), $x_{6}$ (hemo (hemophilia (0 = no, 1 = yes))), $x_{7}$ (homo (homosexual activity (0 = no, 1 = yes))), and $x_{8}$ (karnof (Karnofsky score (on a scale of 0–100))).

*Single-index component*: Continuous covariates $z_{1}$ (CD80 (baseline CD8 count)), $z_{2}$ (CD820 (CD8 count at 20 ± 5 weeks)), $z_{3}$ (CD420 (CD4 count at 20 ± 5 weeks)), $z_{4}$ (CD40 (baseline CD4 count)), $z_{5}$ (wtkg (weight)), and $z_{6}$ (age (age (yrs) at baseline)).

**Figure 1:**
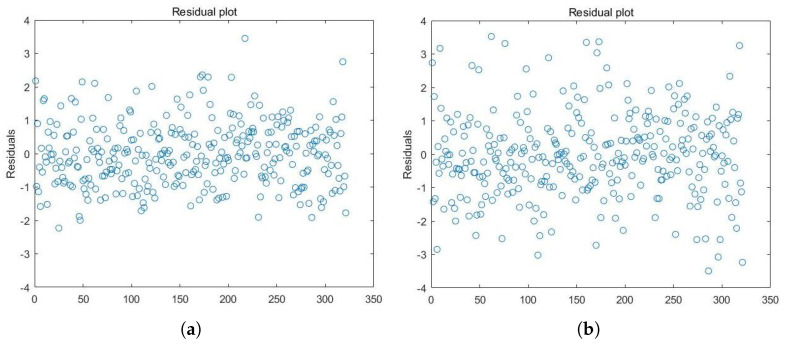
Residual plots for the PLSIM. (**a**) Residual plot of PEL. (**b**) Residual plot of PVS.

After standardizing *Y*, we specify the following heteroscedastic PLSIM:

$$Y=\theta^{⊤}X+g\left(Z^{⊤}\gamma \right)+\varepsilon ,$$

where $X^{⊤}=\left(x_{1},\cdots ,x_{8}\right)$ and $Z^{⊤}=\left(z_{1},\cdots ,z_{6}\right)$. The test for heteroscedasticity confirms that the model is homoscedastic. We applied the Epanechnikov kernel function in this data analysis. According to Condition 2, the bandwidth was set to $n^{-1/5}$, which means that h≈0.32. We required $\gamma ={\left(\gamma_{1},\cdots ,\gamma_{6}\right)}^{⊤}$ to have unit length to ensure identifiability. We compared our results to the PVS method in [16], and the results are summarized in Table 5, with residual plots shown in Figure 1. The residual sums of squares estimated by PEL and PVS were 186 and 194, respectively. From these results, homosexuality exhibits a significant positive linear association with *Y*. The estimated coefficient for antiretroviral therapy (str2) is negative, indicating a beneficial effect of this treatment for asymptomatic patients with HIV. CD820 shows a negative nonlinear relationship with *Y*, while age, CD40, and CD420 are positively associated with *Y*. These factors play important roles in antiretroviral regimens. Moreover, both methods yield similar results, but the approach in [16] produces a slightly larger standard error.

## Figures and Tables

**Table 1:** Variable selection results for the PEL method.

(p,r)	*n*	Kernel Function	Mean Count of Zero Coefficients	
			Correct	Incorrect
(10,10)	50	Epanechnikov	(4.47 [55.9%], 3.82 [54.6%])	(2.51, 3.24)
		Cosine	(4.35 [54.4%], 3.69 [52.7%])	(2.38, 3.47)
(10,10)	100	Epanechnikov	(6.93 [86.6%], 5.63 [80.4%])	(0.52, 0.76)
		Cosine	(6.86 [85.8%], 5.58 [79.7%])	(0.55, 0.74)
(10,10)	200	Epanechnikov	(7.32 [91.5%], 6.27 [89.5%])	(0.34, 0.51)
		Cosine	(7.29 [91.1%], 6.24 [89.1%])	(0.37, 0.58)
(20,20)	50	Epanechnikov	(9.71 [53.9%], 8.95 [52.6%])	(4.83, 5.95)
		Cosine	(9.58 [53.2%], 8.79 [51.7%])	(4.92, 6.14)
(20,20)	100	Epanechnikov	(12.69 [70.5%], 11.81 [69.4%])	(3.58, 5.32)
		Cosine	(12.66 [70.3%], 11.67 [68.6%])	(3.71, 5.45)
(20,20)	200	Epanechnikov	(15.81 [87.8%], 14.71 [86.5%])	(0.42, 0.87)
		Cosine	(15.28 [84.9%], 14.56 [85.6%])	(0.45, 0.93)

**Table 2:** Estimation mean and standard deviations of the PEL estimators (the values in parentheses are the corresponding standard deviations).

(p,r)	*n*	Kernel Function	$\theta_{1}$	$\theta_{\left(p-1\right)}$	$\gamma_{2}$	$\gamma_{3}$
(10,10)	50	Epanechnikov	4.35 (2.616)	2.74 (1.738)	3.91 (2.237)	−4.96 (3.521)
		Cosine	4.27 (2.853)	2.59 (1.802)	4.14 (2.394)	−4.58 (3.475)
(10,10)	100	Epanechnikov	1.94 (0.128)	0.96 (0.106)	1.56 (0.132)	−1.93 (0.103)
		Cosine	2.06 (0.131)	0.94 (0.114)	1.43 (0.146)	−2.09 (0.115)
(10,10)	200	Epanechnikov	1.95 (0.087)	1.03 (0.082)	1.56 (0.105)	−2.04 (0.103)
		Cosine	2.02 (0.094)	1.06 (0.091)	1.43 (0.113)	−2.07 (0.107)
(20,20)	50	Epanechnikov	4.92 (3.587)	3.17 (3.264)	5.89 (4.316)	−6.83 (4.763)
		Cosine	4.53 (3.951)	2.96 (3.728)	6.34 (4.512)	−6.71 (4.625)
(20,20)	100	Epanechnikov	2.97 (1.438)	1.53 (0.896)	2.16 (1.048)	−2.79 (0.951)
		Cosine	3.18 (1.502)	1.61 (0.925)	2.12 (1.073)	−2.85 (1.027)
(20,20)	200	Epanechnikov	2.09 (0.135)	1.05 (0.107)	1.54 (0.139)	−1.96 (0.114)
		Cosine	2.12 (0.139)	1.07 (0.119)	1.43 (0.147)	−2.08 (0.121)

**Table 3:** Variable selection results for the PEL and PVS methods.

(p,r)	*n*	Method	Mean Count of Zero Coefficients	
			Correct	Incorrect
(20,20)	200	PEL	(15.21 [84.5%], 13.45 [84.1%])	(0.37, 1.03)
		PVS	(14.75 [81.9%], 13.17 [82.3%])	(0.45, 1.26)
	400	PEL	(15.89 [88.3%], 14.02 [87.6%])	(0.35, 0.85)
		PVS	(15.46 [85.8%], 13.74 [85.9%])	(0.41, 0.92)
(20,30)	200	PEL	(15.24 [84.6%], 22.56 [86.7%])	(0.39, 1.27)
		PVS	(14.98 [83.2%], 22.12 [85.1%])	(0.38, 1.35)
	400	PEL	(15.79 [87.7%], 23.31 [89.7%])	(0.34, 1.14)
		PVS	(15.13 [84.1%], 22.85 [87.9%])	(0.43, 1.28)
(30,20)	200	PEL	(24.38 [87.1%], 13.51 [84.4%])	(0.38, 1.24)
		PVS	(23.85 [85.2%], 12.97 [81.1%])	(0.44, 1.31)
	400	PEL	(25.14 [89.7%], 14.13 [88.3%])	(0.33, 1.17)
		PVS	(24.73 [88.3%], 13.54 [84.6%])	(0.39, 1.26)
(30,30)	200	PEL	(24.25 [86.6%], 23.36 [89.8%])	(0.40, 1.31)
		PVS	(23.72 [84.7%], 22.68 [87.2%])	(0.47, 1.38)
	400	PEL	(25.27 [90.0%], 23.15 [89.1%])	(0.38, 1.19)
		PVS	(24.54 [87.6%], 22.82 [87.7%])	(0.45, 1.25)

**Table 4:** Estimation mean and standard deviations of the PEL and PVS estimators (the Values in parentheses are the corresponding standard deviations).

(p,r)	*n*	Method	$\theta_{1}$	$\theta_{\left(p-1\right)}$	$\gamma_{2}$	$\gamma_{3}$	$\gamma_{4}$
(20,20)	200	PEL	1.05 (0.132)	−1.04 (0.101)	1.06 (0.143)	2.07 (0.127)	0.95 (0.106)
		PVS	1.12 (0.145)	−1.08 (0.134)	0.93 (0.175)	2.11 (0.142)	1.08 (0.131)
	400	PEL	0.98 (0.102)	−1.03 (0.091)	1.04 (0.126)	1.98 (0.108)	1.06 (0.113)
		PVS	1.07 (0.134)	−1.06 (0.114)	0.96 (0.135)	2.08 (0.125)	1.05 (0.117)
(20,30)	200	PEL	0.94 (0.137)	−1.05 (0.109)	1.10 (0.128)	2.06 (0.121)	1.07 (0.149)
		PVS	1.86 (0.153)	−1.12 (0.142)	0.92 (0.136)	1.96 (0.147)	1.13 (0.151)
	400	PEL	0.96 (0.125)	−1.04 (0.098)	1.08 (0.124)	2.03 (0.119)	1.09 (0.128)
		PVS	1.91 (0.138)	−1.09 (0.117)	0.92 (0.225)	1.95 (0.124)	1.92 (0.137)
(30,20)	200	PEL	1.09 (0.141)	−1.13 (0.135)	1.07 (0.127)	1.94 (0.130)	1.08 (0.143)
		PVS	0.89 (0.159)	−1.08 (0.162)	1.09 (0.143)	1.91 (0.129)	1.13 (0.156)
	400	PEL	1.06 (0.098)	−0.96 (0.107)	1.03 (0.126)	2.05 (0.115)	1.04 (0.112)
		PVS	1.86 (0.116)	−1.58 (0.154)	0.94 (0.139)	1.97 (0.129)	1.07 (0.123)
(30,30)	200	PEL	1.14 (0.129)	−1.13 (0.138)	0.91 (0.142)	2.09 (0.138)	1.12 (0.128)
		PVS	0.79 (0.148)	−1.18 (0.145)	0.88 (0.157)	1.90 (0.141)	0.93 (0.135)
	400	PEL	1.09 (0.114)	−1.07 (0.126)	0.93 (0.119)	2.10 (0.128)	1.09 (0.119)
		PVS	1.15 (0.123)	−1.15 (0.131)	1.05 (0.125)	1.89 (0.133)	0.92 (0.121)

**Table 5:** Estimations and confidence intervals of the methods of PEL and PVS (the values in parentheses are the corresponding confidence intervals).

Variable	Method	
	PEL	PVS
homo	0.193 ([0.065, 0.317])	0.131 ([0.001, 0.262])
str2	−0.322 ([−0.517, −0.016])	−0.045 ([−0.324, 0.234])
age	0.528 ([0.314, 0.735])	0.153 ([−0.118, 0.424])
CD820	−0.691 ([−0.478, −0.893])	−0.208 ([−0.413, 0.003])
cD40	0.255 ([0.009, 0.452])	0.446 ([0.134, 0.758])
cD420	0.423 ([0.126, 0.674])	0.857 ([0.536, 1.178])

## Data Availability

Data are contained within the article.

## Appendix A

Let $D_{n}=\{{\left(\theta^{⊤},\gamma^{⊤}\right)}^{⊤}:∥{\left(\theta^{⊤},\gamma^{⊤}\right)}^{⊤}-{\left(\theta_{0}^{⊤},\gamma_{0}^{⊤}\right)}^{⊤}∥\leq ca_{n}\}$ with a positive constant *C*, $a_{n}=O_{p}\left\{{\left(p/n\right)}^{1/2}\right\}$, and let $∥A∥={\left\{tr\left(A^{⊤}A\right)\right\}}^{\frac{1}{2}}$ denote the Frobenius norm for *A*.

We present some lemmas before proving Theorems as follows.

**Lemma** **A1.**
*Let $∥\tilde{\theta }-\theta ∥=O_{p}\left(a_{n}\right)$ and $∥\tilde{\gamma }-\gamma ∥=O_{p}\left(a_{n}\right)$. Under Conditions 1–9, we have*

$$\underset{\eta \in R}{\sup}\left|\hat{w}\left(\eta \right)-w\left(\eta \right)\right|=O_{p}\left\{h_{2}^{2}+\log n{\left(p/n\right)}^{1/2}h_{2}^{-1/2}\right\},$$

*where*

$$\hat{w}\left(\eta \right)=\sum_{i=1}^{n}K_{h_{2}}\left(\eta_{i}-\eta \right)/\sum_{i=1}^{n}K_{h_{2}}\left(\eta_{i}-\eta \right)e_{i}^{2},$$

$$e_{i}=Y_{i}-{\tilde{\theta }}^{⊤}X_{i}-\tilde{g}\left(Z_{i}^{⊤}\tilde{\gamma }\right)={\left(\theta -\tilde{\theta }\right)}^{⊤}X_{i}+\tilde{g}\left(Z_{i}^{⊤}\gamma \right)-\tilde{g}\left(Z_{i}^{⊤}\tilde{\gamma }\right)+g\left(Z_{i}^{⊤}\gamma \right)-\tilde{g}\left(Z_{i}^{⊤}\gamma \right)+\varepsilon_{i}$$

*and*

$$\tilde{g}\left(Z_{i}^{⊤}\gamma \right)={\left(n-1\right)}^{-1}\sum_{j\neq i}K_{h}\left(Z_{j}^{⊤}\gamma -Z_{i}^{⊤}\gamma \right)Y_{i}.$$

**Proof** **of** **Lemma** **A1.** According to Lemma 3.1 and Lemma 3.3 in Zhu and Fang [31], we have

(A1) $$\underset{(Z_{i}^{⊤}\gamma )\in R}{\sup}\left|g\left(Z_{i}^{⊤}\gamma \right)-\tilde{g}\left(Z_{i}^{⊤}\gamma \right)\right|=O_{p}\left\{h_{2}^{2}+{\left(p/n\right)}^{1/2}h_{2}^{-1/2}\log n\right\}$$

and

(A2) $$\underset{\eta \in R}{\sup}\left|\frac{\sum_{i=1}^{n}K_{h_{2}}\left(\eta_{i}-\eta \right)\varepsilon_{i}^{2}}{\sum_{i=1}^{n}K_{h_{2}}\left(\eta_{i}-\eta \right)}-\frac{1}{w(\eta )}\right|=O_{p}\left\{h_{2}^{2}+{\left(p/n\right)}^{1/2}h_{2}^{-1/2}\log n\right\}.$$

Combining (20) and (21), we can obtain that

(A3) $$\begin{array}{cc} \underset{\eta \in R}{\sup} & \left|\sum_{i=1}^{n}K_{h_{2}}\left(\eta_{i}-\eta \right){\left\{g\left(Z_{i}^{⊤}\gamma \right)-\tilde{g}\left(Z_{i}^{⊤}\gamma \right)\right\}}^{2}/\sum_{i=1}^{n}K_{h_{2}}\left(\eta_{i}-\eta \right)\right|\\ \; & =O_{p}\left\{h_{2}^{4}+\frac{p\log^{2}n}{\left(nh_{2}\right)}\right\}. \end{array}$$

Because of $∥\tilde{\theta }-\theta ∥=O_{p}\left(a_{n}\right)$ and $E(X_{i}^{2}|\eta_{i})<\infty$, we have

(A4) $$\underset{\eta \in R}{\sup}\left|\sum_{i=1}^{n}K_{h_{2}}\left(\eta_{i}-\eta \right){\left\{X_{i}^{⊤}\left(\tilde{\theta }-\theta \right)\right\}}^{2}/\sum_{i=1}^{n}K_{h_{2}}\left(\eta_{i}-\eta \right)\right|=O_{p}\left(p/n\right).$$

Using Lemma 1 of Ma and Zhu [6], we obtain that

(A5) $$\underset{Z_{i}\in R^{p}}{\sup}\underset{\left\{\tilde{\gamma }:∥\tilde{\gamma }-\gamma ∥\leq a_{n}\right\}}{\sup}\left|\left\{\tilde{g}\left(Z_{i}^{⊤}\gamma \right)-\tilde{g}\left(Z_{i}^{⊤}\tilde{\gamma }\right)\right\}-E\left\{\tilde{g}\left(Z_{i}^{⊤}\gamma \right)-\tilde{g}\left(Z_{i}^{⊤}\tilde{\gamma }\right)\right\}\right|=o_{p}\left(a_{n}\right).$$

By (24) and $∥\tilde{\gamma }-\gamma ∥=O_{p}\left(a_{n}\right)$, together with Taylor’s expansion, we can show that

(A6) $$\begin{array}{cc} \underset{\eta \in R}{\sup} & \left|\sum_{i=1}^{n}K_{h_{2}}\left(\eta_{i}-\eta \right){\left\{\tilde{g}\left(Z_{i}^{⊤}\gamma \right)-\tilde{g}\left(Z_{i}^{⊤}\tilde{\gamma }\right)\right\}}^{2}/\sum_{i=1}^{n}K_{h_{2}}\left(\eta_{i}-\eta \right)\right|\\ \; & =O_{p}\left\{h_{2}^{4}+\frac{p\log^{2}n}{\left(nh_{2}\right)}\right\}. \end{array}$$

Obviously, $n^{-1/2}\sum_{i=1}^{n}\tilde{g}\left(X_{i}\right)\varepsilon_{i}$ can be written as

$$n^{-1/2}\sum_{i=1}^{n}\tilde{g}\left(X_{i}\right)\varepsilon_{i}=n^{-3/2}\sum_{i\neq j}^{n}K_{h}\left(X_{i}-X_{j}\right)\left(\varepsilon_{i}Y_{j}-\varepsilon_{j}Y_{i}\right).$$

Let r(X)=E(Y|X). According to Serfling [32], $n^{-3/2}\sum_{i\neq j}^{n}K_{h}\left(X_{i}-X_{j}\right)\left(\varepsilon_{i}Y_{j}-\varepsilon_{j}Y_{i}\right)$ can be approximated by its projection, which means that

$$\left|n^{-1/2}\sum_{i=1}^{n}\tilde{g}\left(X_{i}\right)\varepsilon_{i}-n^{-1/2}\sum_{i=1}^{n}\varepsilon_{i}E\left\{K_{h}\left(X_{i}-X_{j}\right)r\left(X_{i}\right)|X_{i}\right\}\right|=o_{p}\left(n^{-1}p\log\left(n\right)\right).$$

Therefore, we have

(A7) $$n^{-1/2}\sum_{i=1}^{n}\varepsilon_{i}\left\{\tilde{g}\left(X_{i}\right)-r\left(X_{i}\right)f\left(X_{i}\right)\right\}=o_{p}\left(n^{-1}p\log\left(n\right)\right),$$

where f(X) is the density function of *X*. By combining (20)–(26), we have

$$\underset{\eta \in R}{\sup}\left|\hat{w}\left(\eta \right)-w\left(\eta \right)\right|=O_{p}\left\{h_{2}^{2}+\log n{\left(p/n\right)}^{1/2}h_{2}^{-1/2}\right\}.$$

Thus, Lemma A1 holds. □

**Lemma** **A2.**
*Under Conditions 1–9, we have*

(1) $\frac{1}{\sqrt{n}}\sum_{i=1}^{n}{\hat{\xi }}_{i}\left(\theta ,\gamma \right)=\frac{1}{\sqrt{n}}\sum_{i=1}^{n}\xi_{i}\left(\theta ,\gamma \right)+o_{p}\left(1\right);$
(2) $\frac{1}{n}\sum_{i=1}^{n}{\hat{\xi }}_{i}\left(\theta ,\gamma \right){\hat{\xi }}_{i}^{⊤}\left(\theta ,\gamma \right)=\frac{1}{n}\sum_{i=1}^{n}\xi_{i}\left(\theta ,\gamma \right)\xi_{i}^{⊤}\left(\theta ,\gamma \right)+o_{p}\left(1\right).$

**Proof** **of** **Lemma** **A2.** We first expand the expression as follows:

(A8) $$\begin{array}{cc} w_{i} & \varepsilon_{i}g^{\prime }\left(Z_{i}^{⊤}\gamma \right)\left\{Z_{i}-\frac{E(w_{i}Z_{i}|Z_{i}^{⊤}\gamma )}{E(w_{i}|Z_{i}^{⊤}\gamma )}\right\}-{\hat{w}}_{i}{\tilde{\varepsilon }}_{i}{\hat{g}}^{\prime }\left(Z_{i}^{⊤}\gamma \right)\left\{Z_{i}-\frac{\hat{E}\left({\hat{w}}_{i}Z_{i}|Z_{i}^{⊤}\gamma \right)}{\hat{E}\left({\hat{w}}_{i}|Z_{i}^{⊤}\gamma \right)}\right\}\\ = & \varepsilon_{i}\left(w_{i}-{\hat{w}}_{i}\right)g^{\prime }\left(Z_{i}^{⊤}\gamma \right)\left\{Z_{i}-\frac{E(w_{i}Z_{i}|Z_{i}^{⊤}\gamma )}{E(w_{i}|Z_{i}^{⊤}\gamma )}\right\}\\ \; & +{\hat{w}}_{i}\varepsilon_{i}\left\{g^{\prime }\left(Z_{i}^{⊤}\gamma \right)-{\hat{g}}^{\prime }\left(Z_{i}^{⊤}\gamma \right)\right\}\left\{Z_{i}^{⊤}-\frac{E(w_{i}Z_{i}^{⊤}|Z_{i}^{⊤}\gamma )}{E(w_{i}|Z_{i}^{⊤}\gamma )}\right\}\\ \; & -{\hat{w}}_{i}g^{\prime }\left(Z_{i}^{⊤}\gamma \right)\left\{\hat{g}\left(Z_{i}^{⊤}\gamma \right)-g\left(Z_{i}^{⊤}\gamma \right)\right\}\left\{Z_{i}-\frac{E(w_{i}Z_{i}|Z_{i}^{⊤}\gamma )}{E(w_{i}|Z_{i}^{⊤}\gamma )}\right\}\\ \; & -{\hat{w}}_{i}\left\{\hat{g}\left(Z_{i}^{⊤}\gamma \right)-g\left(Z_{i}^{⊤}\gamma \right)\right\}\left\{g^{\prime }\left(Z_{i}^{⊤}\gamma \right)-{\hat{g}}^{\prime }\left(Z_{i}^{⊤}\gamma \right)\right\}\left\{Z_{i}^{⊤}-\frac{E(w_{i}Z_{i}^{⊤}|Z_{i}^{⊤}\gamma )}{E(w_{i}|Z_{i}^{⊤}\gamma )}\right\}\\ \; & +{\hat{w}}_{i}\varepsilon_{i}{\hat{g}}^{\prime }\left(Z_{i}^{⊤}\gamma \right)\left(\frac{\hat{E}\left({\hat{w}}_{i}Z_{i}|Z_{i}^{⊤}\gamma \right)}{\hat{E}\left({\hat{w}}_{i}|Z_{i}^{⊤}\gamma \right)}-\frac{E(w_{i}Z_{i}^{⊤}|Z_{i}^{⊤}\gamma )}{E(w_{i}|Z_{i}^{⊤}\gamma )}\right)\\ \; & -{\hat{w}}_{i}{\hat{g}}^{\prime }\left(Z_{i}^{⊤}\gamma \right)\left\{\hat{g}\left(Z_{i}^{⊤}\gamma \right)-g\left(Z_{i}^{⊤}\gamma \right)\right\}\left(\frac{\hat{E}\left({\hat{w}}_{i}Z_{i}|Z_{i}^{⊤}\gamma \right)}{\hat{E}\left({\hat{w}}_{i}|Z_{i}^{⊤}\gamma \right)}-\frac{E(w_{i}Z_{i}^{⊤}|Z_{i}^{⊤}\gamma )}{E(w_{i}|Z_{i}^{⊤}\gamma )}\right)\\ = & \frac{1}{\sqrt{n}}\left(\sum_{i=1}^{n}A_{1i}+\sum_{i=1}^{n}A_{2i}+\sum_{i=1}^{n}A_{3i}+\sum_{i=1}^{n}A_{4i}+\sum_{i=1}^{n}A_{5i}+\sum_{i=1}^{n}A_{6i}\right), \end{array}$$

and

(A9) $$\begin{array}{cc} w_{i}\varepsilon_{i} & \left(X_{i}-\frac{E(w_{i}Z_{i}|Z_{i}^{⊤}\gamma )}{E(w_{i}|Z_{i}^{⊤}\gamma )}\right)-{\hat{w}}_{i}{\tilde{\varepsilon }}_{i}\left(X_{i}-\frac{\hat{E}\left({\hat{w}}_{i}X_{i}|Z_{i}^{⊤}\gamma \right)}{\hat{E}\left({\hat{w}}_{i}|Z_{i}^{⊤}\gamma \right)}\right)\\ = & \varepsilon_{i}\left(w_{i}-{\hat{w}}_{i}\right)\left(X_{i}-\frac{E(w_{i}Z_{i}|Z_{i}^{⊤}\gamma )}{E(w_{i}|Z_{i}^{⊤}\gamma )}\right)\\ \; & +{\hat{w}}_{i}\left\{\hat{g}\left(Z_{i}^{⊤}\gamma \right)-g\left(Z_{i}^{⊤}\gamma \right)\right\}\left(X_{i}-\frac{E(w_{i}X_{i}|Z_{i}^{⊤}\gamma )}{E(w_{i}|Z_{i}^{⊤}\gamma )}\right)\\ \; & +{\hat{w}}_{i}\varepsilon_{i}\left(\frac{\hat{E}\left({\hat{w}}_{i}X_{i}|Z_{i}^{⊤}\gamma \right)}{\hat{E}\left({\hat{w}}_{i}|Z_{i}^{⊤}\gamma \right)}-\frac{E(w_{i}Z_{i}|Z_{i}^{⊤}\gamma )}{E(w_{i}|Z_{i}^{⊤}\gamma )}\right)\\ \; & +{\hat{w}}_{i}\left(\frac{\hat{E}\left({\hat{w}}_{i}X_{i}|Z_{i}^{⊤}\gamma \right)}{\hat{E}\left({\hat{w}}_{i}|Z_{i}^{⊤}\gamma \right)}-\frac{E(w_{i}Z_{i}|Z_{i}^{⊤}\gamma )}{E(w_{i}|Z_{i}^{⊤}\gamma )}\right)\left\{g\left(Z_{i}^{⊤}\gamma \right)-\hat{g}\left(Z_{i}^{⊤}\gamma \right)\right\}\\ = & \frac{1}{\sqrt{n}}\left(\sum_{i=1}^{n}A_{7i}+\sum_{i=1}^{n}A_{8i}+\sum_{i=1}^{n}A_{9i}+\sum_{i=1}^{n}A_{10i}\right). \end{array}$$

By Lemma A1, we can obtain

$$∥\sum_{i=1}^{n}A_{1i}∥\leq \sum_{i=1}^{n}\left|(w_{i}-{\hat{w}}_{i})\right|\left|\varepsilon_{i}g^{\prime }\left(Z_{i}^{⊤}\gamma \right)\right|∥\left\{Z_{i}-\frac{E(w_{i}Z_{i}|Z_{i}^{⊤}\gamma )}{E(w_{i}|Z_{i}^{⊤}\gamma )}\right\}∥=o_{p}\left(\sqrt{n}\right),$$

and

$$∥\sum_{i=1}^{n}A_{7i}∥=\sum_{i=1}^{n}\left|(w_{i}-{\hat{w}}_{i})\right|∥\varepsilon_{i}\left(X_{i}-\frac{E(w_{i}Z_{i}|Z_{i}^{⊤}\gamma )}{E(w_{i}|Z_{i}^{⊤}\gamma )}\right)∥=o_{p}\left(\sqrt{n}\right).$$

Write $\varepsilon_{i}=w_{i}g^{\prime }\left(Z_{i}^{⊤}\gamma \right)\left\{Z_{i}-\frac{E(w_{i}Z_{i}|Z_{i}^{⊤}\gamma )}{E(w_{i}|Z_{i}^{⊤}\gamma )}\right\}$. It implies that $E(\varepsilon_{i}|Z_{i}^{⊤}\gamma )=0$. According to Lemma A1 and (26), we have

$$\begin{array}{cc} \; & ∥\sum_{i=1}^{n}\left\{{\hat{w}}_{i}-w_{i}\right\}\left\{\hat{g}\left(Z_{i}^{⊤}\gamma \right)-g\left(Z_{i}^{⊤}\gamma \right)\right\}g^{\prime }\left(Z_{i}^{⊤}\gamma \right)\left\{Z_{i}-\frac{E(w_{i}Z_{i}|Z_{i}^{⊤}\gamma )}{E(w_{i}|Z_{i}^{⊤}\gamma )}\right\}∥\\ \leq & \underset{1\leq i\leq n}{\sup}\left|{\hat{w}}_{i}-w_{i}\right|\sum_{i=1}^{n}\left|\left\{\hat{g}\left(Z_{i}^{⊤}\gamma \right)-g\left(Z_{i}^{⊤}\gamma \right)\right\}\right|∥g^{\prime }\left(Z_{i}^{⊤}\gamma \right)\left\{Z_{i}-\frac{E(w_{i}Z_{i}|Z_{i}^{⊤}\gamma )}{E(w_{i}|Z_{i}^{⊤}\gamma )}\right\}∥\\ = & o_{p}\left(\sqrt{n}\right), \end{array}$$

and

$$∥\sum_{i=1}^{n}w_{i}g^{\prime }\left(Z_{i}^{⊤}\gamma \right)\left\{\hat{g}\left(Z_{i}^{⊤}\gamma \right)-g\left(Z_{i}^{⊤}\gamma \right)\right\}\left\{Z_{i}-\frac{E(w_{i}Z_{i}|Z_{i}^{⊤}\gamma )}{E(w_{i}|Z_{i}^{⊤}\gamma )}\right\}∥=o_{p}\left(\sqrt{n}\right).$$

Thus, $∥\sum_{i=1}^{n}A_{2i}∥=o_{p}\left(\sqrt{n}\right)$. Similarly, we let $\varepsilon_{i}=w_{i}\left\{Z_{i}-\frac{E(w_{i}Z_{i}|Z_{i}^{⊤}\gamma )}{E(w_{i}|Z_{i}^{⊤}\gamma )}\right\}$. We can obtain that $E\left(\varepsilon_{i}|Z_{i}^{⊤}\gamma \right)=0$. According to Lemma A1 and (26) again, we have

$$\begin{array}{cc} ∥\sum_{i=1}^{n}A_{8i}∥\;\leq & \sum_{i=1}^{n}\left|{\hat{w}}_{i}-w_{i}\right|\left|\left\{\hat{g}\left(Z_{i}^{⊤}\gamma \right)-g\left(Z_{i}^{⊤}\gamma \right)\right\}\right|∥\left(X_{i}-\frac{E({\hat{w}}_{i}X_{i}|Z_{i}^{⊤}\gamma )}{E({\hat{w}}_{i}|Z_{i}^{⊤}\gamma )}\right)∥\\ \; & +\sum_{i=1}^{n}\left|\left\{\hat{g}\left(Z_{i}^{⊤}\gamma \right)-g\left(Z_{i}^{⊤}\gamma \right)\right\}\right|∥w_{i}\left(X_{i}-\frac{E({\hat{w}}_{i}X_{i}|Z_{i}^{⊤}\gamma )}{E({\hat{w}}_{i}|Z_{i}^{⊤}\gamma )}\right)∥\\ = & o_{p}\left(\sqrt{n}\right). \end{array}$$

Similarly to Lemma 3 in Ma and Zhu [6], we can show that,

(A10) $$\underset{X}{\sup}\left|n^{-1}\sum_{i=1}^{n}K_{h}\left(X_{i}-X\right)Y_{i}-r\left(X\right)f\left(X\right)\right|=O_{p}\left\{h^{2}+\log n{\left(p/n\right)}^{1/2}h^{-1/2}\right\}.$$

According to Lemma A1, together with (29), we can obtain

(A11) $$∥{\hat{g}}^{\prime }\left(Z_{i}^{⊤}\gamma \right)-g^{\prime }\left(Z_{i}^{⊤}\gamma \right)∥=o_{p}\left(1\right),$$

(A12) $$∥\hat{E}\left\{\hat{w}\left(X,Z\right)Z|\gamma^{⊤}Z_{i}\right\}-E\left\{w\left(X,Z\right)Z|\gamma^{⊤}Z_{i}\right\}∥=o_{p}\left(1\right),$$

(A13) $$∥\hat{E}\left\{\hat{w}\left(X,Z\right)|\gamma^{⊤}Z_{i}\right\}-E\left\{w\left(X,Z\right)|\gamma^{⊤}Z_{i}\right\}∥=o_{p}\left(1\right),$$

and

(A14) $$∥\hat{E}\left\{\hat{w}\left(X,Z\right)X|\gamma^{⊤}Z_{i}\right\}-E\left\{w\left(X,Z\right)X|\gamma^{⊤}Z_{i}\right\}∥=o_{p}\left(1\right).$$

Similarly, to prove $∥\sum_{i=1}^{n}A_{2i}∥=o_{p}\left(\sqrt{n}\right)$, together with (30)–(33), we have $∥\sum_{i=1}^{n}A_{4i}∥$$=o_{p}\left(\sqrt{n}\right)$, $∥\sum_{i=1}^{n}A_{6i}∥=o_{p}\left(\sqrt{n}\right)$, and $∥\sum_{i=1}^{n}A_{10i}∥=o_{p}\left(\sqrt{n}\right)$. Applying (26), together with (31), it is easy to show that $∥\sum_{i=1}^{n}A_{3i}∥=o_{p}\left(\sqrt{n}\right)$. In addition, by combining (28)–(33) and using Lemma A1, we can show that $∥\sum_{i=1}^{n}A_{5i}∥=o_{p}\left(\sqrt{n}\right)$ and $∥\sum_{i=1}^{n}A_{9i}∥=o_{p}\left(\sqrt{n}\right)$.

$$\begin{array}{c} \frac{1}{\sqrt{n}}\left\{\sum_{i=1}^{n}{\hat{\xi }}_{i}\left(\theta ,\gamma \right)-\sum_{i=1}^{n}\xi_{i}\left(\theta ,\gamma \right)\right\}=\frac{1}{\sqrt{n}}\sum_{i=1}^{n}\left(\begin{array}{c} A_{1i}+A_{2i}+A_{3i}+A_{4i}+A_{5i}+A_{6i}\\ A_{7i}+A_{8i}+A_{9i}+A_{10i} \end{array}\right). \end{array}$$

Therefore, we have

$$\frac{1}{\sqrt{n}}\sum_{i=1}^{n}{\hat{\xi }}_{i}\left(\theta ,\gamma \right)=\frac{1}{\sqrt{n}}\sum_{i=1}^{n}\xi_{i}\left(\theta ,\gamma \right)+o_{p}\left(1\right).$$

Next, we will prove the second part of Lemma A2. By Conditions 8 and 9, ∀ϵ>0,

$$\begin{array}{cc} P\{\max_{1\leq i\leq n}∥\xi_{i}\left(\theta ,\gamma \right)∥\;\leq n^{1/4}\sqrt{p}\epsilon \} & \leq \sum_{i=1}^{n}P\{∥\xi_{i}\left(\theta ,\gamma \right)∥\;\leq n^{1/4}\sqrt{p}\epsilon \}\\ \; & \leq \frac{1}{np^{2}\epsilon^{4}}\sum_{i=1}^{n}E{∥\xi_{i}\left(\theta ,\gamma \right)∥}^{4}\\ \; & =\frac{1}{\epsilon^{k}}E{∥\xi_{1}\left(\theta ,\gamma \right)/\sqrt{p}∥}^{4}. \end{array}$$

By Cauchy–Schwarz inequality, we gain that $∥\xi_{1}\left(\theta ,\gamma \right)/\sqrt{p}{∥}^{4}\leq 1/\left(p+r\right)\sum_{l=1}^{p+r}{\left|\xi_{1l}\left(\theta ,\gamma \right)\right|}^{4}$, where $\xi_{1l}\left(\theta ,\gamma \right)$ is the *l*-th component of $\xi_{1}\left(\theta ,\gamma \right)$. This implies that

(A15) $$\max_{1\leq i\leq n}∥\xi_{i}\left(\theta ,\gamma \right)∥=o_{p}\left(n^{1/4}\sqrt{p}\right).$$

$$\begin{array}{cc} \frac{1}{n}\sum_{i=1}^{n}{\hat{\xi }}_{i}\left(\theta ,\gamma \right){\hat{\xi }}_{i}{\left(\theta ,\gamma \right)}^{⊤}= & \frac{1}{n}\sum_{i=1}^{n}\xi_{i}\left(\theta ,\gamma \right)\xi_{i}{\left(\theta ,\gamma \right)}^{⊤}+\frac{1}{n}\sum_{i=1}^{n}\xi_{i}\left(\theta ,\gamma \right){\left(\begin{array}{c} A_{1i}+\cdots +A_{6i}\\ A_{7i}+\cdots +A_{10i} \end{array}\right)}^{⊤}\\ \; & +\frac{1}{n}\sum_{i=1}^{n}\left(\begin{array}{c} A_{1i}+\cdots +A_{6i}\\ A_{7i}+\cdots +A_{10i} \end{array}\right)\xi_{i}{\left(\theta ,\gamma \right)}^{⊤}\\ \; & +\frac{1}{n}\sum_{i=1}^{n}\left(\begin{array}{c} A_{1i}+\cdots +A_{6i}\\ A_{7i}+\cdots +A_{10i} \end{array}\right){\left(\begin{array}{c} A_{1i}+\cdots +A_{6i}\\ A_{7i}+\cdots +A_{10i} \end{array}\right)}^{⊤}. \end{array}$$

According to (34) and Condition 7, we can obtain $1/\sqrt{n}∥\xi_{i}\left(\theta ,\gamma \right)∥=o_{p}\left(1\right)$. Using the proof of the first part above again, we have

$$\sum_{i=1}^{n}A_{ki}\xi_{i}\left(\theta ,\gamma \right)=o_{p}\left(\sqrt{n}\right),\;\;\;k=1,\cdots ,10.$$

Similarly,

$$\sum_{i=1}^{n}A_{ki}A_{li}=o_{p}\left(\sqrt{n}\right),\;\;\;k,l=1,\cdots ,10.$$

By combining the above equations, we have

$$\frac{1}{n}\sum_{i=1}^{n}{\hat{\xi }}_{i}\left(\theta ,\gamma \right){\hat{\xi }}_{i}{\left(\theta ,\gamma \right)}^{⊤}=\frac{1}{n}\sum_{i=1}^{n}\xi_{i}\left(\theta ,\gamma \right)\xi_{i}{\left(\theta ,\gamma \right)}^{⊤}+o_{p}\left(1\right).$$

Therefore, the second part of Lemma A2 holds. □

**Lemma** **A3.**
*Under Conditions 1–9, $∥S_{n}-V∥=O_{p}\left(p/\sqrt{n}\right)$, where $S_{n}=1/n\sum_{i=1}^{n}\xi_{i}\left(\theta ,\gamma \right)\xi_{i}{\left(\theta ,\gamma \right)}^{⊤}$.*

**Proof** **of** **Lemma** **A3.** We can obtain that $tr\left\{{\left(S_{n}-V\right)}^{2}\right\}=O_{p}\left(p^{2}/n\right)$, using Lemma 4 in Chen et al. [23]. Therefore, $∥S_{n}-V∥={\left\{tr\left[{\left(S_{n}-V\right)}^{⊤}\left(S_{n}-V\right)\right]\right\}}^{1/2}=O_{p}\left(pn^{-1/2}\right)$. □

**Lemma** **A4.**
*Under Conditions 1–11, $\max_{1\leq i\leq n}∥{\hat{\xi }}_{i}\left(\theta ,\gamma \right)∥=o_{p}\left(n^{1/4}\sqrt{p}\right)$ and $\max_{1\leq i\leq n}\left|\lambda^{⊤}{\hat{\xi }}_{i}\left(\theta ,\gamma \right)\right|$$=o_{p}\left(1\right)$ for all $\lambda =O_{p}\left(a_{n}\right)$.*

**Proof** **of** **Lemma** **A4.** Based on the proof of the first part in Lemma A2, it is easy to show that

$$∥{\hat{\xi }}_{i}\left(\theta ,\gamma \right)∥=∥\xi_{i}\left(\theta ,\gamma \right)∥+o_{p}\left(1\right).$$

Therefore, by combining the above equation and (34), we have

$$∥{\hat{\xi }}_{i}\left(\theta ,\gamma \right)∥==o_{p}\left(n^{1/4}\sqrt{p}\right),$$

and for all $\lambda =O_{p}\left(a_{n}\right)$,

$$\max_{1\leq i\leq n}\left|\lambda^{⊤}{\hat{\xi }}_{i}\left(\theta ,\gamma \right)\right|=o_{p}\left(1\right).$$

□

**Lemma** **A5.**
*Under Conditions 1–11, $∥\lambda_{\left(\theta_{0},\gamma_{0}\right)}∥=O_{p}\left(a_{n}\right)$ and $∥\lambda_{\left(\hat{\theta },\hat{\gamma }\right)}∥=O_{p}\left(a_{n}\right)$.*

**Proof** **of** **Lemma** **A5.** Let $\lambda_{\left(\theta ,\gamma \right)}=\rho \alpha$, where $\rho =∥\lambda_{\left(\theta ,\gamma \right)}∥$, $\alpha \in R^{p+r}$, and ∥α∥=1. According to (8), $\lambda_{\left(\theta ,\gamma \right)}\in R^{p+r}$ satisfies

$$0=\frac{1}{n}\sum_{i=1}^{n}\frac{{\hat{\xi }}_{i}\left(\theta ,\gamma \right)}{1+\lambda_{\left(\theta ,\gamma \right)}^{⊤}{\hat{\xi }}_{i}\left(\theta ,\gamma \right)}=:\psi \left(\lambda \right).$$

Using $1/\left(1+\lambda_{\left(\theta ,\gamma \right)}^{⊤}{\hat{\xi }}_{i}\left(\theta ,\gamma \right)\right)=1-\lambda_{\left(\theta ,\gamma \right)}^{⊤}{\hat{\xi }}_{i}\left(\theta ,\gamma \right)/\left(1+\lambda_{\left(\theta ,\gamma \right)}^{⊤}{\hat{\xi }}_{i}\left(\theta ,\gamma \right)\right)$, we can obtain that

$$n^{-1}\left|\alpha^{⊤}\sum_{i=1}^{n}{\hat{\xi }}_{i}\left(\theta ,\gamma \right)\right|\geq \frac{\rho }{1+\rho \max_{1\leq i\leq n}∥{\hat{\xi }}_{i}\left(\theta ,\gamma \right)∥}\alpha^{⊤}{\hat{S}}_{n}\left(\theta ,\gamma \right)\alpha ,$$

where ${\hat{S}}_{n}\left(\theta ,\gamma \right)=\frac{1}{n}\sum_{i=1}^{n}{\hat{\xi }}_{i}\left(\theta ,\gamma \right){\hat{\xi }}_{i}{\left(\theta ,\gamma \right)}^{⊤}$. Note that

$$0<1+\lambda_{\left(\theta ,\gamma \right)}^{⊤}{\hat{\xi }}_{i}\left(\theta ,\gamma \right)\leq 1+\rho \max_{1\leq i\leq n}∥{\hat{\xi }}_{i}\left(\theta ,\gamma \right)∥,$$

and we have

(A16) $$\rho \left\{\alpha^{⊤}{\hat{S}}_{n}\left(\theta ,\gamma \right)\alpha -\alpha^{⊤}{\hat{\xi }}_{i}\left(\theta ,\gamma \right)\max_{1\leq i\leq n}∥{\hat{\xi }}_{i}\left(\theta ,\gamma \right)∥\right\}\leq n^{-1}\left|\alpha^{⊤}\sum_{i=1}^{n}{\hat{\xi }}_{i}\left(\theta ,\gamma \right)\right|.$$

Using Lemma A4, we can show

$$n^{-1}|\alpha^{⊤}\sum_{i=1}^{n}{\hat{\xi }}_{i}\left(\theta_{0},\gamma_{0}\right)|\leq n^{-1}∥\sum_{i=1}^{n}{\hat{\xi }}_{i}\left(\theta_{0},\gamma_{0}\right)∥=O_{p}\left(a_{n}\right).$$

Therefore,

(A17) $$\max_{1\leq i\leq n}n^{-1}∥{\hat{\xi }}_{i}\left(\theta_{0},\gamma_{0}\right)∥|\alpha^{⊤}\sum_{i=1}^{n}{\hat{\xi }}_{i}\left(\theta_{0},\gamma_{0}\right)|=o_{p}\left(1\right).$$

Combining (35) and (36), we have

$$|\rho \left\{\alpha^{⊤}{\hat{S}}_{n}\left(\theta_{0},\gamma_{0}\right)\alpha +o_{p(1)}\right\}|=O_{p}\left(a_{n}\right).$$

Using Lemma A1, we can show that $P(\alpha^{⊤}{\hat{S}}_{n}\left(\theta_{0},\gamma_{0}\right)\alpha \geq \frac{1}{2}C)\to 1$ as n→∞. Therefore, $\rho =O_{p}\left(a_{n}\right)$, which means that

$$∥\lambda_{\left(\theta_{0},\gamma_{0}\right)}∥=\rho =O_{p}\left(a_{n}\right),$$

and the proof of $∥\lambda_{\left(\hat{\theta },\hat{\gamma }\right)}∥=O_{p}\left(a_{n}\right)$ follows by Owen [33]. □

**Lemma** **A6.**
*Under Conditions 1–11, ${\tilde{\ell }}_{p}\left(\theta ,\gamma \right)$ attains its minimum in $D_{n}$, with probability approaching 1.*

**Proof** **of** **Lemma** **A6.** The proof of Lemma A6 is analogous to that of Lemma 2 in Tang and Leng [24], and is therefore omitted for brevity. □

**Proof** **of** **Theorem** **1.** According to Lemma A6, ${\tilde{\ell }}_{p}\left(\theta ,\gamma \right)$ attains its minimum in $D_{n}$. Let ${\left(\theta^{⊤},\gamma^{⊤}\right)}^{⊤}\in D_{n}$. combining Lemma A2 and Taylor’s expansion, we can show that

$$\begin{array}{cc} \frac{1}{n}\frac{\partial {\tilde{\ell }}_{p}\left(\theta ,\gamma \right)}{\partial \theta_{j}}= & \frac{1}{n}\sum_{i=1}^{n}\frac{\lambda^{⊤}\partial {\hat{\xi }}_{i}\left(\theta ,\gamma \right)/\partial \theta_{j}}{1+\lambda^{⊤}{\hat{\xi }}_{i}\left(\theta ,\gamma \right)}+p_{\nu }^{\prime }\left(\right|\theta_{j}\left|\right)\mathrm{sign}\left(\theta_{j}\right)\\ = & \frac{1}{n}\sum_{i=1}^{n}\frac{\lambda^{⊤}\partial \xi_{i}\left(\theta ,\gamma \right)/\partial \theta_{j}}{1+\lambda^{⊤}\xi_{i}\left(\theta ,\gamma \right)}+o_{p}\left(1\right)+p_{\nu }^{\prime }\left(\right|\theta_{j}\left|\right)\mathrm{sign}\left(\theta_{j}\right)\\ = & \frac{1}{n}\sum_{i=1}^{n}\lambda^{⊤}\left\{\frac{\partial \xi_{i}\left(\theta_{0},\gamma_{0}\right)}{\partial \theta_{j}}+\frac{\partial^{2}\xi_{i}\left(\theta_{0},\gamma_{0}\right)}{\partial \theta_{j}\partial \theta^{⊤}}\left(\theta -\theta_{0}\right)\right\}\\ \; & +o_{p}\left(1\right)+p_{\nu }^{\prime }\left(\right|\theta_{j}\left|\right)\mathrm{sign}\left(\theta_{j}\right)\\ = & A_{1}+A_{2}+p_{\nu }^{\prime }\left(\right|\theta_{j}\left|\right)\mathrm{sign}\left(\theta_{j}\right)+o_{p}\left(1\right), \end{array}$$

and

$$\begin{array}{cc} \frac{1}{n}\frac{\partial {\tilde{\ell }}_{p}\left(\theta ,\gamma \right)}{\partial \gamma_{j}}= & \frac{1}{n}\sum_{i=1}^{n}\frac{\lambda^{⊤}\partial {\hat{\xi }}_{i}\left(\theta ,\gamma \right)/\partial \gamma_{j}}{1+\lambda^{⊤}{\hat{\xi }}_{i}\left(\theta ,\gamma \right)}+p_{\nu }^{\prime }\left(\right|\gamma_{j}\left|\right)\mathrm{sign}\left(\gamma_{j}\right)\\ = & \frac{1}{n}\sum_{i=1}^{n}\frac{\lambda^{⊤}\partial \xi_{i}\left(\theta ,\gamma \right)/\partial \gamma_{j}}{1+\lambda^{⊤}\xi_{i}\left(\theta ,\gamma \right)}+o_{p}\left(1\right)+p_{\nu }^{\prime }\left(\right|\gamma_{j}\left|\right)\mathrm{sign}\left(\gamma_{j}\right)\\ = & \frac{1}{n}\sum_{i=1}^{n}\lambda^{⊤}\left\{\frac{\partial \xi_{i}\left(\theta_{0},\gamma_{0}\right)}{\partial \gamma_{j}}+\frac{\partial^{2}\xi_{i}\left(\theta_{0},\gamma_{0}\right)}{\partial \gamma_{j}\partial \gamma^{⊤}}\left(\gamma -\gamma_{0}\right)\right\}\\ \; & +o_{p}\left(1\right)+p_{\nu }^{\prime }\left(\right|\gamma_{j}\left|\right)\mathrm{sign}\left(\gamma_{j}\right)\\ = & A_{3}+A_{4}+p_{\nu }^{\prime }\left(\right|\gamma_{j}\left|\right)\mathrm{sign}\left(\gamma_{j}\right)+o_{p}\left(1\right). \end{array}$$

It follows from Conditions 5 and 6 and Lemma A5 that

$$\begin{array}{cc} \max_{j\notin A_{1}}\left(\right|A_{1}\left|\right) & =\max_{j\notin A_{1}}\frac{1}{n}\left|\sum_{i=1}^{n}\lambda^{⊤}\left\{E\frac{\partial \xi_{i}\left(\theta_{0},\gamma_{0}\right)}{\partial \theta_{j}}+\left(\frac{\partial \xi_{i}\left(\theta_{0},\gamma_{0}\right)}{\partial \theta_{j}}-E\frac{\partial \xi_{i}\left(\theta_{0},\gamma_{0}\right)}{\partial \theta_{j}}\right)\right\}\right|\\ \; & \leq \max_{j\notin A_{1}}\left|\lambda^{⊤}E\frac{\partial \xi (\theta_{0},\gamma_{0})}{\partial \theta_{j}}\right|+\frac{1}{n}∥\lambda^{⊤}∥∥\sum_{i=1}^{n}\left(\frac{\partial \xi_{i}\left(\theta_{0},\gamma_{0}\right)}{\partial \theta_{j}}-E\frac{\partial \xi (\theta_{0},\gamma_{0})}{\partial \theta_{j}}\right)∥\\ \; & =o_{p}\left(1\right), \end{array}$$

and

$$\begin{array}{cc} \max_{j\notin A_{1}}\left(\right|A_{2}\left|\right) & \leq \frac{1}{n}∥\lambda^{⊤}∥∥\sum_{i=1}^{n}\left(\frac{\partial^{2}\xi_{i}\left(\theta_{0},\gamma_{0}\right)}{\partial \theta_{j}\partial \theta^{⊤}}-E\frac{\partial^{2}\xi \left(\theta_{0},\gamma_{0}\right)}{\partial \theta_{j}\partial \theta^{⊤}}\right)∥∥\left(\theta -\theta_{0}\right)∥\\ \; & +\max_{j\notin A_{1}}\left|\lambda^{⊤}E\frac{\partial^{2}\xi \left(\theta_{0},\gamma_{0}\right)}{\partial \theta_{j}\partial \theta^{⊤}}\right|∥\left(\theta -\theta_{0}\right)∥=o_{p}\left(1\right). \end{array}$$

Similarly, we can obtain

$$\begin{array}{cc} \max_{j\notin A_{2}}\left(\right|A_{3}\left|\right) & =\max_{j\notin A_{2}}\frac{1}{n}\left|\sum_{i=1}^{n}\lambda^{⊤}\left\{E\frac{\partial \xi_{i}\left(\theta_{0},\gamma_{0}\right)}{\partial \gamma_{j}}+\left(\frac{\partial \xi_{i}\left(\theta_{0},\gamma_{0}\right)}{\partial \gamma_{j}}-E\frac{\partial \xi_{i}\left(\theta_{0},\gamma_{0}\right)}{\partial \gamma_{j}}\right)\right\}\right|\\ \; & =o_{p}\left(1\right), \end{array}$$

and

$$\begin{array}{cc} \max_{j\notin A_{2}}\left(\right|A_{4}\left|\right) & \leq \frac{1}{n}∥\lambda^{⊤}∥∥\sum_{i=1}^{n}\left(\frac{\partial^{2}\xi_{i}\left(\theta_{0},\gamma_{0}\right)}{\partial \gamma_{j}\partial \gamma^{⊤}}-E\frac{\partial^{2}\xi \left(\theta_{0},\gamma_{0}\right)}{\partial \gamma_{j}\partial \gamma^{⊤}}\right)∥∥\left(\gamma -\gamma_{0}\right)∥\\ \; & +\max_{j\notin A_{2}}\left|\lambda^{⊤}E\frac{\partial^{2}\xi \left(\theta_{0},\gamma_{0}\right)}{\partial \gamma_{j}\partial \gamma^{⊤}}\right|∥\left(\gamma -\gamma_{0}\right)∥=o_{p}\left(1\right). \end{array}$$

Based on Condition 10, we can show that $P_{\tau }^{\prime }\left(\right|\theta_{j}\left|\right)\mathrm{sign}{\left(\theta_{j}\right)}_{\left\{j\notin A_{1}\right\}}=\tau$ and $P_{\tau }^{\prime }\left(\right|\theta_{j}\left|\right)\mathrm{sign}{\left(\theta_{j}\right)}_{\left\{j\notin A_{1}\right\}}$$=\tau \mathrm{sign}{\left(\theta_{j}\right)}_{\left\{j\notin A_{1}\right\}}$. Thus, with probability approaching 1, $\partial {\tilde{l}}_{p}\left(\theta ,\gamma \right)/\partial \theta_{j}$ is dominated by the sign of $\theta_{j}$, $j\notin A_{1}$, as n→∞. It means that $\lim_{n\to \infty }P\left({\hat{\theta }}_{2}=0\right)=1$. Similarly, using Condition 10 again, we have $P_{\nu }^{\prime }\left(\right|\gamma_{j}\left|\right)\mathrm{sign}{\left(\gamma_{j}\right)}_{\left\{j\notin A_{2}\right\}}=\nu$, $P_{\nu }^{\prime }\left(\right|\gamma_{j}\left|\right)\mathrm{sign}{\left(\gamma_{j}\right)}_{\left\{j\notin A_{2}\right\}}=\nu \mathrm{sign}{\left(\gamma_{j}\right)}_{\left\{j\notin A_{2}\right\}}$, and $\lim_{n\to \infty }P\left({\hat{\gamma }}_{2}=0\right)=1$. Therefore, Theorem 1 (1) is proved. In this section, we begin to prove Theorem 1 (2). Minimising (9) is equal to minimising the following function

$$\begin{array}{cc} {\tilde{\ell }}_{p}\left(\theta ,\gamma ,\lambda ,\mu ,\vartheta \right)= & \frac{1}{n}\sum_{i=1}^{n}\log\left\{1+\lambda^{⊤}{\hat{\xi }}_{i}\left(\theta ,\gamma \right)\right\}+\sum_{i=1}^{p}p_{\tau }\left(\right|\theta_{i}\left|\right)+\sum_{i=1}^{r}p_{\nu }\left(\right|\gamma_{i}\left|\right)\\ \; & +\mu^{⊤}H_{2}\theta +\vartheta^{⊤}H_{4}\gamma , \end{array}$$

where μ and ϑ are also Lagrange multipliers. Define

$$\begin{array}{cc} Q_{1n}\left(\theta ,\gamma ,\lambda ,\mu ,\vartheta \right) & =\frac{1}{n}\sum_{i=1}^{n}\frac{{\hat{\xi }}_{i}\left(\theta ,\gamma \right)}{1+\lambda^{⊤}{\hat{\xi }}_{i}\left(\theta ,\gamma \right)},\\ Q_{2n}\left(\theta ,\gamma ,\lambda ,\mu ,\vartheta \right) & =\frac{1}{n}\sum_{i=1}^{n}\frac{{\left\{\partial {\hat{\xi }}_{i}\left(\theta ,\gamma \right)/\partial \theta \right\}}^{⊤}\lambda }{1+\lambda^{⊤}{\hat{\xi }}_{i}\left(\theta ,\gamma \right)}+b_{1}\left(\theta \right)+H_{2}^{⊤}\mu ,\\ Q_{3n}\left(\theta ,\gamma ,\lambda ,\mu ,\vartheta \right) & =\frac{1}{n}\sum_{i=1}^{n}\frac{{\left\{\partial {\hat{\xi }}_{i}\left(\theta ,\gamma \right)/\partial \gamma \right\}}^{⊤}\lambda }{1+\lambda^{⊤}{\hat{\xi }}_{i}\left(\theta ,\gamma \right)}+b_{2}\left(\gamma \right)+H_{4}^{⊤}\vartheta ,\\ Q_{4n}\left(\theta ,\gamma ,\lambda ,\mu ,\vartheta \right) & =H_{2}\theta ,Q_{5n}\left(\theta ,\gamma ,\lambda ,\mu ,\vartheta \right)=H_{4}\gamma , \end{array}$$

where

$$b_{1}\left(\theta \right)=\{P_{\tau }^{\prime }\left(\right|\theta_{1}|)\mathrm{sign}\left(\theta_{1}\right),\cdots ,P_{\tau }^{\prime }\left(\right|\theta_{p_{1}}|)\mathrm{sign}\left(\theta_{p_{1}}\right)\},$$

and

$$b_{2}\left(\gamma \right)=\{P_{\nu }^{\prime }\left(\right|\gamma_{1}|)\mathrm{sign}\left(\gamma_{1}\right),\cdots ,P_{\nu }^{\prime }\left(\right|\gamma_{p_{2}}|)\mathrm{sign}\left(\theta_{p_{2}}\right)\}.$$

The minimizer $\left(\hat{\theta },\hat{\gamma },\hat{\lambda },\hat{\mu },\hat{\vartheta }\right)$ satisfies $Q_{jn}\left(\theta ,\gamma ,\lambda ,\mu ,\vartheta \right)=0$, j=1,⋯,n. According to $∥\lambda ∥=O_{p}\left(a_{n}\right)$, $Q_{2n}\left(\theta ,\gamma ,\lambda ,\mu ,\vartheta \right)=0$, and $Q_{3n}\left(\theta ,\gamma ,\lambda ,\mu ,\vartheta \right)=0$, we can obtain $∥\mu ∥=O_{p}\left(a_{n}\right)$ and $∥\vartheta ∥=O_{p}\left(a_{n}\right)$. Thus, by expanding $Q_{jn}\left(\theta ,\gamma ,\lambda ,\mu ,\vartheta \right)$ at $\left(\theta_{0},\gamma_{0},0,0,0\right)$, we have

$$\begin{array}{c} \left(\begin{array}{c} -Q_{1n}\left(\theta_{0},\gamma_{0},0,0,0\right)\\ 0\\ 0\\ 0\\ 0 \end{array}\right)=\left(\begin{array}{ccccc} -{\hat{S}}_{n} & {\hat{M}}_{1} & {\hat{M}}_{2} & 0 & 0\\ {\hat{M}}_{1}^{T} & b_{1}^{\prime }\left(\theta \right) & 0 & H_{2}^{⊤} & 0\\ {\hat{M}}_{2} & 0 & b_{2}^{\prime }\left(\gamma \right) & 0 & H_{4}^{⊤}\\ 0 & H_{2} & 0 & 0 & 0\\ 0 & 0 & H_{4} & 0 & 0 \end{array}\right)\left(\begin{array}{c} \hat{\lambda }-0\\ \hat{\theta }-\theta_{0}\\ \hat{\gamma }-\gamma_{0}\\ \hat{\mu }-0\\ \hat{\vartheta }-0 \end{array}\right), \end{array}$$

where ${\hat{M}}_{1}=n^{-1}\sum_{i=1}^{n}\partial {\hat{\xi }}_{i}\left(\theta ,\gamma \right)/\partial \theta$ and ${\hat{M}}_{2}=n^{-1}\sum_{i=1}^{n}\partial {\hat{\xi }}_{i}\left(\theta ,\gamma \right)/\partial \gamma$. Let $M_{1}=n^{-1}\sum_{i=1}^{n}\partial \xi_{i}\left(\theta ,\gamma \right)/\partial \theta$ and $M_{2}=n^{-1}\sum_{i=1}^{n}\partial {\hat{\xi }}_{i}\left(\theta ,\gamma \right)/\partial \gamma$, it is easy to show that

(A18) $$\begin{array}{c} \left(\begin{array}{c} -Q_{1n}\left(\theta_{0},\gamma_{0},0,0,0\right)\\ 0\\ 0\\ 0\\ 0 \end{array}\right)=\left(\begin{array}{ccccc} -S_{n} & M_{1} & M_{2} & 0 & 0\\ M_{1}^{T} & 0 & 0 & H_{2}^{⊤} & 0\\ M_{2} & 0 & 0 & 0 & H_{4}^{⊤}\\ 0 & H_{2} & 0 & 0 & 0\\ 0 & 0 & H_{4} & 0 & 0 \end{array}\right)\left(\begin{array}{c} \hat{\lambda }-0\\ \hat{\theta }-\theta_{0}\\ \hat{\gamma }-\gamma_{0}\\ \hat{\mu }-0\\ \hat{\vartheta }-0 \end{array}\right)+R_{n}, \end{array}$$

where $R_{n}=\sum_{k=1}^{8}R_{n}^{\left(k\right)}$, $R_{n}^{\left(1\right)}={\left(R_{1n}^{⊤(1)},R_{2n}^{⊤(1)},R_{3n}^{⊤(1)},0,0\right)}^{⊤}$, $R_{1n}^{\left(1\right)}\in R^{p+r-1}$, $R_{2n}^{\left(1\right)}\in R^{p}$, $R_{3n}^{\left(1\right)}\in R^{r-1}$, the *k*-th component of $R_{jn}\left(1\right)$ is given by

$$R_{jn,k}\left(1\right)=\frac{1}{2}{\left(\hat{\phi }-\phi \right)}^{⊤}\frac{\partial^{2}Q_{jn,k}\left(\phi^{*}\right)}{\partial \phi \partial \phi^{⊤}}\left(\hat{\phi }-\phi \right),$$

$\phi ={\left(\lambda^{⊤},\theta^{⊤},\gamma^{⊤}\right)}^{⊤}$, $\phi^{*}={\left(\lambda^{*⊤},\theta^{*⊤},\gamma^{*⊤}\right)}^{⊤}$ such that $∥\lambda^{*}∥\leq ∥\hat{\lambda }∥$, $∥\theta^{*}-\theta_{0}∥\leq ∥\hat{\theta }-\theta_{0}∥$, and $∥\gamma^{*}-\gamma_{0}∥\leq ∥\hat{\gamma }-\gamma_{0}∥$. In addition, we have $R_{n}^{\left(2\right)}={\left\{0,b_{1}^{⊤}\left(\theta_{0}\right),0,0,0\right\}}^{⊤}$, $R_{n}^{\left(3\right)}={\left\{0,0,b_{2}^{⊤}\left(\gamma_{0}\right),0,0\right\}}^{⊤}$, $R_{n}^{\left(4\right)}={\left\{0,{\left\{b_{1}^{\prime }\left(\theta^{*}\right)\left(\theta^{*}-\theta_{0}\right)\right\}}^{⊤},0,0,0\right\}}^{⊤}$, $R_{n}^{\left(5\right)}={\left\{0,0,{\left\{b_{2}^{\prime }\left(\gamma^{*}\right)\left(\gamma^{*}-\gamma_{0}\right)\right\}}^{⊤},0,0\right\}}^{⊤}$, $R_{n}^{\left(6\right)}={\left\{{\left\{\left({\hat{S}}_{n}\left(\theta_{0},\gamma_{0}\right)-S_{n}\right)\hat{\lambda }\right\}}^{⊤}+{\left\{{\hat{M}}_{1}\left(\theta_{0},\gamma_{0}\right)\left(\hat{\theta }-\theta_{0}\right)\right\}}^{⊤}+{\left\{{\hat{M}}_{2}\left(\theta_{0},\gamma_{0}\right)\left(\hat{\gamma }-\gamma_{0}\right)\right\}}^{⊤},0,0,0,0\right\}}^{⊤}$, $R_{n}^{\left(7\right)}={\left\{0,{\left\{{\left({\hat{M}}_{1}\left(\theta_{0},\gamma_{0}\right)-M_{1}\right)}^{⊤}\hat{\lambda }\right\}}^{⊤},0,0,0\right\}}^{⊤}$, and $R_{n}^{\left(8\right)}=\{0,0,\{({\hat{M}}_{2}(\theta_{0}$, $\gamma_{0})-M_{2}{)}^{⊤}\hat{\lambda }{{\}}^{⊤},0,0\}}^{⊤}$. By Conditions 5–7 and Lemma A5, we can show that

$$∥R_{ln}^{\left(1\right)}{∥}^{2}\leq n^{-2}{∥\hat{\phi }-\phi ∥}^{4}\sum_{i,j,k=1}^{2(p+r-1)}\frac{\partial^{2}Q_{i}\left(X,Z\right)}{\partial \phi_{j}\partial \phi_{k}}=O_{p}\left\{{\left(p+r-1\right)}^{3}a_{n}^{4}\right\},l=1,2,3.$$

Combining this with Condition 7, it implies that $∥R_{n}^{\left(1\right)}∥=o_{p}\left(1/\sqrt{n}\right)$. According to Conditions 10 and 11, it easy to show that $R_{n}^{\left(2\right)}=o_{p}\left(1/\sqrt{n}\right)$, $R_{n}^{\left(3\right)}=o_{p}\left(1/\sqrt{n}\right)$, $R_{n}^{\left(4\right)}=o_{p}\left(1/\sqrt{n}\right)$, and $R_{n}^{\left(5\right)}=o_{p}\left(1/\sqrt{n}\right)$. Similarly to Leng and Tang [25], we can also prove that $∥R_{n}^{\left(6\right)}∥=o_{p}\left(1/\sqrt{n}\right)$, $∥R_{n}^{\left(7\right)}∥=o_{p}\left(1/\sqrt{n}\right)$, and $∥R_{n}^{\left(8\right)}∥=o_{p}\left(1/\sqrt{n}\right)$. Therefore, we have $∥R_{n}∥=o_{p}\left(1/\sqrt{n}\right)$. Let

$$\psi ={\left(\theta^{⊤},\gamma^{⊤},\mu^{⊤},\vartheta^{⊤}\right)}^{⊤},\;\;\;K_{11}=-S_{n},\;\;\;K_{12}=\left(M_{1},M_{2},0,0\right),\;\;\;K_{21}=K_{12}^{⊤}$$

and

$$\begin{array}{c} K_{22}=\left(\begin{array}{cccc} 0 & 0 & H_{2}^{⊤} & 0\\ 0 & 0 & 0 & H_{4}^{⊤}\\ H_{2} & 0 & 0 & 0\\ 0 & H_{4} & 0 & 0 \end{array}\right),\;\;\;\;K=\left(\begin{array}{cc} K_{11} & K_{12}\\ K_{21} & K_{22} \end{array}\right). \end{array}$$

By inverting (37), it can be shown that

(A19) $${\left({\left(\hat{\lambda }-0\right)}^{⊤},{\left(\hat{\psi }-\psi \right)}^{⊤}\right)}^{⊤}=K^{-1}\left\{{\left(-Q_{1n}{\left(\theta_{0},\gamma_{0},0,0,0\right)}^{⊤},0\right)}^{⊤}+o_{p}(n^{-1/2})\right\}.$$

Applying block matrix inversion, we have

$$\begin{array}{c} K^{-1}=\left(\begin{array}{cc} K_{11}^{-1}+K_{11}^{-1}K_{12}F^{-1}K_{21}K_{11}^{-1} & -K_{11}^{-1}K_{12}F^{-1}\\ -F^{-1}K_{21}K_{11}^{-1} & F^{-1} \end{array}\right), \end{array}$$

where

$$F=K_{22}-K_{21}K_{11}^{-1}K_{12}.$$

Thus,

$$\hat{\psi }-\psi =F^{-1}K_{21}K_{11}^{-1}Q_{1n}\left(\theta ,\gamma ,\lambda ,\mu ,\vartheta \right)+o_{p}\left(n^{-1/2}\right),$$

and

$$\begin{array}{c} F^{-1}=\left(\begin{array}{cc} U^{-1}-\Omega & U^{-1}H^{⊤}{\left(HU^{-1}H^{⊤}\right)}^{-1}\\ {\left(HU^{-1}H^{⊤}\right)}^{-1}HU^{-1} & -{\left(HU^{-1}H^{⊤}\right)}^{-1} \end{array}\right), \end{array}$$

where $\Omega =U^{-1}H^{⊤}{\left(HU^{-1}H^{⊤}\right)}^{-1}HU^{-1}$ and

$$\begin{array}{c} U=\left(\begin{array}{cc} M_{1}^{⊤}S_{n}^{-1}M_{1} & M_{1}^{⊤}S_{n}^{-1}M_{2}\\ M_{2}^{⊤}S_{n}^{-1}M_{1} & M_{2}^{⊤}S_{n}^{-1}M_{2} \end{array}\right). \end{array}$$

This implies that

$$\begin{array}{c} \left(\begin{array}{c} \hat{\theta }-\theta_{0}\\ \hat{\gamma }-\gamma_{0} \end{array}\right)=\left\{U^{-1}-\Omega \right\}\left\{\frac{1}{n}\sum_{i=1}^{n}\xi_{i}\left(\theta_{0},\gamma_{0}\right)+o_{p}\left(n^{-\frac{1}{2}}\right)\right\}. \end{array}$$

Furthermore, we can obtain

$$\begin{array}{c} \left(\begin{array}{c} {\hat{\theta }}_{1}-\theta_{10}\\ {\hat{\gamma }}_{1}-\gamma_{10} \end{array}\right)=H_{0}\left\{U^{-1}-\Omega \right\}\left\{\frac{1}{n}\sum_{i=1}^{n}\xi_{i}\left(\theta_{0},\gamma_{0}\right)+o_{p}\left(n^{-\frac{1}{2}}\right)\right\}. \end{array}$$

Using Lemma A3, we have $\mathrm{Var}\left\{n^{1/2}{\left({\hat{\theta }}_{1}^{⊤},{\hat{\gamma }}_{1}^{⊤}\right)}^{⊤}\right\}=I_{B}=H_{0}U^{-1}VU^{-1}H_{0}^{⊤}-H_{0}U^{-1}H^{⊤}$${\left(HU^{-1}H^{⊤}\right)}^{-1}H_{2}U^{-1}VA^{-1}H_{2}^{⊤}{\left(HU^{-1}H^{⊤}\right)}^{-1}HU^{-1}H_{0}^{⊤}$. Define $Y_{ni}=\frac{1}{\sqrt{n}}T_{ni}$, where $T_{ni}=BI_{B}^{-\frac{1}{2}}\left(H_{0}U^{-1}-H_{0}U^{-1}H^{⊤}{\left(HU^{-1}H^{⊤}\right)}^{-1}HU^{-1}\right)\xi_{i}\left(\theta_{0},\gamma_{0}\right)$. According to the central limit theorem, it follows that

$$\frac{1}{\sqrt{n}}BI_{B}^{-\frac{1}{2}}\left\{H_{0}U^{-1}-H_{0}U^{-1}H^{⊤}{\left(HU^{-1}H^{⊤}\right)}^{-1}HU^{-1}\right\}\sum_{i=1}^{n}\xi_{i}\left(\theta_{0},\gamma_{0}\right)\to N\left(0,G\right)$$

in distribution. □

**Proof** **of** **Theorem** **2.** Define ${\hat{\varphi }}_{i}={\hat{\lambda }}^{⊤}{\hat{\xi }}_{i}\left(\hat{\theta },\hat{\gamma }\right)$ and $\varphi_{i}={\hat{\lambda }}^{⊤}\xi_{i}\left(\hat{\theta },\hat{\gamma }\right)$ for i=1,⋯,n. Combining Lemma A2, Lemma A4, and Taylor’s expansion, we can show that

(A20) $$\begin{array}{cc} {\tilde{l}}_{p}\left(\hat{\theta },\hat{\gamma }\right) & =\sum_{i=1}^{n}\left\{{\hat{\varphi }}_{i}-\frac{{\hat{\varphi }}_{i}^{2}}{2}+\frac{{\hat{\varphi }}_{i}^{3}}{3{\left(1+\zeta_{i}\right)}^{4}+o_{p}\left(1\right)}\right\}\\ \; & =\sum_{i=1}^{n}\left\{\varphi_{i}-\frac{\varphi_{i}^{2}}{2}+\frac{\varphi_{i}^{3}}{3{\left(1+\zeta_{i}\right)}^{4}+o_{p}\left(1\right)}\right\}+o_{p}\left(1\right), \end{array}$$

where $|\zeta_{i}\left|\leq \right|\varphi_{i}|$. According to (38), the asymptotic expansion for $\hat{\lambda }$ can be shown as

$$\hat{\lambda }=\left\{\Sigma^{-1}+\Sigma^{-1}\left(M_{1},M_{2}\right)F_{1}{\left(M_{1},M_{2}\right)}^{⊤}\Sigma^{-1}\right\}\left({\bar{\xi }}_{i}+o_{p}\left(1\right)\right),$$

where $F_{1}=U^{-1}-\Omega$, $\Omega =U^{-1}H^{⊤}{\left(HU^{-1}H^{⊤}\right)}^{-1}HU^{-1}$, and ${\bar{\xi }}_{i}=\frac{1}{n}\sum_{i=1}^{n}\xi_{i}\left(\theta_{0},\gamma_{0}\right)$. From (39), we can gain the expansion of ${\tilde{l}}_{p}\left(\hat{\theta },\hat{\gamma }\right)$ as follows

$${\tilde{l}}_{p}\left(\hat{\theta },\hat{\gamma }\right)=n{\bar{\xi }}_{i}^{⊤}\Sigma^{-1}\left(M_{1},M_{2}\right)U^{-1}H^{⊤}{\left(HU^{-1}H^{⊤}\right)}^{-1}HU^{-1}{\left(M_{1},M_{2}\right)}^{⊤}\Sigma^{-1}{\bar{\xi }}_{i}+o_{p}\left(1\right).$$

There exist values of ${\tilde{H}}_{2}$ and ${\tilde{H}}_{4}$ that satisfy ${\tilde{H}}_{2}\theta =0$, ${\tilde{H}}_{4}\theta =0$, ${\tilde{H}}_{2}{\tilde{H}}_{2}^{⊤}=I_{p-q_{1}}$, and ${\tilde{H}}_{4}{\tilde{H}}_{4}^{⊤}=I_{p-q_{2}}$. Let $\tilde{H}=\left(\begin{array}{cc} {\tilde{H}}_{2} & 0\\ 0 & {\tilde{H}}_{4} \end{array}\right)$. Similarly, we can show that

$$\begin{array}{c} {\tilde{l}}_{p}{\left(\hat{\theta },\hat{\gamma }\right)}_{L_{n}(\theta_{0}^{⊤},L_{n}{\left(\gamma_{0}^{⊤}\right)}^{⊤}=0)}=n{\bar{\xi }}_{i}^{⊤}\Sigma^{-1}\left(M_{1},M_{2}\right)\tilde{\Omega }{\left(M_{1},M_{2}\right)}^{⊤}\Sigma^{-1}{\bar{\xi }}_{i}+o_{p}\left(1\right), \end{array}$$

where $\tilde{\Omega }=U^{-1}{\tilde{H}}^{⊤}{\left(\tilde{H}U^{-1}{\tilde{H}}^{⊤}\right)}^{-1}\tilde{H}U^{-1}$. By combining above two equations, it follows that

$${\tilde{l}}_{p}\left(L_{n}\right)=n{\bar{\xi }}_{i}^{⊤}\Sigma^{-1/2}\left(P_{1}-P_{2}\right)\Sigma^{-1/2}{\bar{\xi }}_{i}+o_{p}\left(1\right),$$

where $P_{1}=\Sigma^{-1/2}\left(M_{1},M_{2}\right)\tilde{\Omega }{\left(M_{1},M_{2}\right)}^{⊤}\Sigma^{-1/2},$ $P_{2}=\Sigma^{-1/2}\left(M_{1},M_{2}\right)\Omega {\left(M_{1},M_{2}\right)}^{⊤}\Sigma^{-1/2}$. Because $P_{1}-P_{2}$ is an idempotent matrix with rank $q_{1}+q_{2}$, $P_{1}-P_{2}$ can be denoted as $P_{n}^{⊤}P_{n}$, where $P_{n}\in R^{\left(q_{1}+q_{2}\right)\times \left(p+r-1\right)}$ and $P_{n}P_{n}^{⊤}=I_{q_{1}+q_{2}}$. By the central limit theorem, we have $\sqrt{n}P_{n}\Sigma^{-1/2}{\bar{\xi }}_{i}\overset{L}{\to }N\left(0,I_{q_{1}+q_{2}}\right)$. Therefore, ${\tilde{l}}_{p}\left(L_{n}\right)\overset{L}{\to }\chi_{q_{1}+q_{2}}^{2}$ and Theorem 2 follows. □

The SIM and PLM are two special cases of the PLSIM. According to the same arguments in the proofs of Theorems 1 and 2, Corollaries 1–4 can be proved in a similar manner, and are hence omitted.
